# Suppression of Cell Tumorigenicity by Non-neural Pro-differentiation Factors via Inhibition of Neural Property in Tumorigenic Cells

**DOI:** 10.3389/fcell.2021.714383

**Published:** 2021-09-14

**Authors:** Xiaoli Yang, Ning Cao, Lu Chen, Lin Liu, Min Zhang, Ying Cao

**Affiliations:** ^1^Shenzhen Research Institute of Nanjing University, Shenzhen, China; ^2^MOE Key Laboratory of Model Animals for Disease Study, Model Animal Research Center, Nanjing University, Nanjing, China; ^3^Jiangsu Key Laboratory of Molecular Medicine of Medical School, Nanjing University, Nanjing, China

**Keywords:** neural ground state, neural stemness, non-neural pro-differentiation factor, tumorigenicity, tumorigenesis, AOM/DSS model of colitis-associated cancer, differentiation, tumor suppression

## Abstract

Our studies have demonstrated that cell tumorigenicity and pluripotent differentiation potential stem from neural stemness or a neural ground state, which is defined by a regulatory network of higher levels of machineries for basic cell physiological functions, including cell cycle, ribosome biogenesis, protein translation, spliceosome, epigenetic modification factors, reprogramming factors, etc., in addition to the neural stemness specific factors. These machineries and neural stemness factors mostly play cancer-promoting roles. It can be deduced that differentiation requires the repression of neural ground state and causes the reduction or loss of neural ground state and thus tumorigenicity in tumorigenic cells. Formerly, we showed that neuronal differentiation led to reduced tumorigenicity in tumorigenic cells. In the present study, we show that non-neural pro-differentiation factors, such as GATA3, HNF4A, HHEX, and FOXA3 that specify mesodermal or/and endodermal tissues during vertebrate embryogenesis, suppress tumorigenicity via repression of neural stemness and promotion of non-neural property in tumorigenic cells. Mechanistically, these transcription factors repress the transcription of neural enriched genes and meanwhile activate genes that specify non-neural properties via direct binding to the promoters of these genes. We also show that combined expression of HHEX and FOXA3 suppresses tumorigenesis effectively in the AOM/DSS model of colitis-associated cancer. We suggest that targeting the property of neural stemness could be an effective strategy for cancer therapy.

## Introduction

The enormous genetic and phenotypic heterogeneity of tumors has caused difficulties in understanding tunorigenesis and effective clinical therapy of cancers. Our studies have demonstrated that cell tumorigenicity stems from neural stemness. This is because cells capable of tumor formation exhibit the property of neural stem/progenitor cells (NSCs/NPCs) and share the regulatory networks with NSCs/NPCs (Cao, 2017; Zhang et al., 2017; Lei et al., 2019; Xu et al., 2021). Importantly, among different cell types except embryonic stem cells (ESCs) and induced pluripotent stem cells (iPS), NSCs/NPCs show tumorigenic potential when transplanted into immunodeficient nude mice subcutaneously or via tail vein and the ability of differentiation into different tissue or cell types from all germ layers (Xu et al., 2021). Moreover, tumor cells capable of tumor formation also show the potential of differentiation of different cell types derived from three germ layers (Xu et al., 2021). Together with the evolutionary advantage of neural state and other studies in developmental biology, tumor biology and evolution, in particular the neural default model of embryonic pluripotent cells, we proposed that neural stemness represents the ground state of tumorigenic and differentiation potential (Cao, 2020). This means that tumorigenesis resembles a process of severely chaotic and degenerated embryonic tissue differentiation, which is driven by cells with the property of neural stemness (Cao, 2017, 2020; Xu et al., 2021). There has been evidence that neural progenitor cells (NPCs) promote tumor growth and metastasis (Mauffrey et al., 2019), and gain of neural stemness in intestinal stem cells drives tumorigenesis (Li Z. et al., 2020).

Neural stemness as the ground state (the neural ground state) of tumorigenicity and differentiation potential is reflected by that many machineries required for basic physiological functions of cells and factors involved in developmental programs are enriched in embryonic neural cells or NSCs, such as those for cell cycle, ribosome biogenesis, proteasome assembly, protein translation, spliceosome, epigenetic modification, reprogramming, etc. (Cao, 2020). These machineries or factors, most of which are upregulated in and promote tumorigenesis, work together to define a cell state of fast cell cycle and high proliferation with the potential of pluripotent differentiation. Therefore, loss of neural stemness via neuronal differentiation leads to a reduction or loss of cell tumorigenicity (Zhang et al., 2017; Xu et al., 2021). High expression level of the genes for the basic machineries and factors in neural cells and low level in non-neural cells during embryogenesis suggest that non-neural differentiation requires repression of neural enriched genes/factors. As a result, non-neural differentiation should cause a reduction or loss of neural stemness and hence tumorigenicity. In the present study, we tested the effect of non-neural pro-differentiation factors, GATA3, HNF4A, HHEX, or FOXA3, on cell tumorigenicity. Gata3 is selectively expressed in trophoblast in mouse blastocyst, induces trophoblast cell fate in ESCs, and drives trophoblast differentiation in trophoblast stem cells (Ralston et al., 2010). Hnf4a is an endoderm-specific factor during early embryognesis and able to convert fibroblasts into hepatocytes in combination with other factors (Li et al., 2008; Huang et al., 2014). *Hhex* is transcribed and plays extensive roles in the formation of endodermal and mesodermal issues or organs during vertebrate embryogenesis, such as liver, pancreatic, and heart development (Martinez Barbera et al., 2000; Walmsley et al., 2002; Foley and Mercola, 2005; Soufi and Jayaraman, 2008). Like *Hnf4a*, *Foxa3* is also expressed only in embryonic endodermal structures and its protein reprograms fibroblasts into hepatocytes together with other factors (Monaghan et al., 1993; Huang et al., 2011). They are all transcription factors. We observed that enforced expression of these factors in cancer cells or neural stem cells (NSCs) inhibited cell tumorigenicity. The inhibitory effect was achieved by repression of some basic cell physiological machineries that are enriched in cancer cells and NSCs. Combined expression of HHEX and FOXA3 exhibited an inhibitory effect of tumorigenesis in a chemically induced colon cancer model. The result might suggest an alternative strategy for cancer therapy via the suppression effect of non-neural pro-differentiation factors on neural stemness or the neural ground state.

## Materials and Methods

### Cell Culture

HEK293T, U118MG, and HCT116 cells were cultured in Dulbecco’s Modified Eagle’s Medium (DMEM. Thermo Fisher Scientific, #11965-092), SH-SY5Y was cultured in a 1:1 mix of MEM (Gibco, #11090073) and F12 medium, and NE-4C cells were cultured in MEM containing 1% glutamax (Gibco, #35050061) and 1% MEM non-essential amino acids (Thermo Fisher Scientific, #11140050). All culture media were supplemented with 10% fetal bovine serum (FBS, Gibco, #10099141) and with 50 U/ml penicillin and 50 μg/ml streptomycin. NE-4C was cultured in dishes coated with 10 μg/ml poly-D-lysine (PDL, Sigma-Aldrich, #P0899). Cells were cultured at 37°C with 5% CO_2_. Cells were also cultured in defined serum-free medium Ndiff 227 (CellArtis, #Y40002) at 37°C with 5% CO2, which is used for culture of NSCs or cancer cells to form neurospheres or neurosphere-like structures (Xu et al., 2021).

U118MG (Cat. No.: #TCHu216), HCT116 (Cat. No.: #TCHu 99), and NE-4C (#SCSP-1501) were purchased from the Cellbank of Chinese Academy of Sciences (Shanghai, China). SH-SY5Y (ATCC^®^ CRL-2266^TM^) was purchased from ATCC (ATCC, United States). Cancer cell lines were authenticated with short tandem repeat profiling, and cells were detected free of mycoplasma contamination with PCR.

### Plasmid Construction

For enforced expression of HHEX, Foxa3, GATA3, or Hnf4a in cells, the open reading frames (ORFs) of *HHEX* were PCR amplified from the cDNA transcribed from total RNA of HEK293T cells, GATA3 was amplified from human endothelial cells, *Hnf4a* and *Foxa3* were from mouse liver cDNA, and subcloned to lentiviral vector pLVX-IRES-puro and pLVX-IRES-ZsGreen. Four consecutive HA tags were also introduced from the vector pCS2 + 4 × HA to pLVX-IRES-puro to generate HA-tagged HHEX or FOXA3 fusion protein constructs. ORFs of HHEX and Foxa3 were also cloned to the adeno-associated virus (AAV) vector GPAAV-CMV-MCS-EF1-ZsGreen-WPRE to generate AAV-HHEX and AAV-Foxa3 constructs, respectively. Construction of AAV constructs and AAV packaging and titering were performed by Genomeditech (Shanghai, China).

To make luciferase reporters for monitoring gene transcription activity, −373/+43 promoter region of *SOX2* gene (transcription start as + 1), −898/+61 promoter region of *CDKN1A* gene, and −470/+51 promoter region of *SBDS* gene were amplified from genomic DNA of HEK293T cells and subcloned to pGL3-basic vector (Promega) to generate reporters *SOX2*-Luc, *CDKN1A*-Luc, and *SBDS*-Luc, respectively. The primers used for amplifying promoters were (Numbers indicate the position in the genes. The first base of the transcriptional start site is designated as +1.):

SOX2luc-F: ccgg acgcgt CGCGTCCCATCCTCATTTAAGT (−373), SOX2Luc-R: ccgg aagctt TCTGCCTTGACAACTCCT GATAC (+43); CDKN1Aluc-F: ccgg acgcgt CCATGCCCGG CTGATTTTTG (−898), CDKN1Aluc-R: ccgg aagctt CTGTCTC CTACCATCCCCTT (+61); SBDSluc-F: ccgg acgcgt TGAC AGAGTGAGACTGACTT (−470), SBDSluc-R: ccgg aagctt GACTCACTAGCTTCAGGCAG (+51).

### Lentiviral Packaging and Infection

To achieve enforced expression of HHEX, Foxa3, GATA3, or Hnf4a in cells, lentivirus packaging and infection was performed essentially as described (Lei et al., 2019). Virus particles were concentrated by centrifugation at 14,000 rpm at 4°C for 2 h. Infected cells were selected with puromycin at 1 μg/ml. Cells infected with virus particles packaged with the empty lentiviral vector were used as control.

### Immunofluorescence (IF)

Immunofluorescence detection of proteins in cells was performed exactly as described (Xu et al., 2021). Primary antibodies were (Fold of antibody dilution indicated): TUBB3 (Cell Signaling Technology, #4466. 1:1,000), NEFL (Cell Signaling Technology, #2837. 1:2,000), EZH2 (Cell Signaling Technology, #5246. 1:2,000), FOXA3 (Santa Cruz, #sc-74424. 1:500), and HHEX (R&D, #MAB83771. 1:500). Secondary antibodies were donkey anti-mouse or rabbit IgG (H + L) Alexa Flour 594 (Thermo Fisher Scientific, #A21207, #A21203. 1:1,000). Cell nuclei were counterstained with DAPI. After staining, slides were rinsed, and coverslips were mounted with anti-fade mounting medium (Invitrogen, #S36936). Cells were viewed under a fluorescence microscope (Zeiss LSM 880).

### Immunoblotting (IB)

Immunoblotting detection of protein expression was performed with whole cell lysates using the conventional method. Primary antibodies were (Fold of antibody dilution indicated): β-ACT (Cell Signaling Technology, #4970. 1:10,000), AURKA (Cell Signaling Technology, #14475. 1:1,000), CCND1 (Cell Signaling Technology, #2978. 1:1,000), CDH1 (Cell Signaling Technology, #3195. 1:1,000), CDH2 (Cell Signaling Technology, #13116. 1:1,000), DNMT1 (Abcam, #ab13537. 1:1,000), EGFR (Cell Signaling Technology, #4267. 1:1,000), EZH2 (Cell Signaling Technology, #5246. 1:4,000), FOXA3 (Abcam, #108454. 1:2,000), FOXM1 (Cell Signaling Technology, #5436. 1:1,000), G9A (Cell Signaling Technology, #3306. 1:1,000), GATA3 (Cell Signaling Technology, #5852. 1:1,000), HDAC1 (Cell Signaling Technology, #5356. 1:1,000), HHEX (R&D, #MAB83771. 1:2000), Hnf4a (Abclonal, #A13998. 1:1,000), LSD1 (Cell Signaling Technology, #2139. 1:2,000), MSI1 (Cell Signaling Technology, #5663. 1:1,000), MYC (Cell Signaling Technology, #13987. 1:2,000), NEFL (Cell Signaling Technology, #2837. 1:2,000), NEFM (Cell Signaling Technology, #2838. 1:2,000), PCNA (Cell Signaling Technology, #13110. 1:3,000), PDGFRA (Cell Signaling Technology, #5241. 1:2,000), PRMT1 (Cell Signaling Technology, #2449. 1:2,000), SETD1A (Cell Signaling Technology, #61702. 1:1,000), SETDB1 (Cell Signaling Technology, #2196. 1:1,000), SNAI1 (Cell Signaling Technology, #3879. 1:1,000), SNAI2 (Cell Signaling Technology, #9585. 1:2,000), SOX1 (Abcam, #ab109290. 1:1,000), SOX2 (Cell Signaling Technology, #23064. 1:1,000), TUBB3 (Cell Signaling Technology, #5568. 1:2,000), YAP1 (Cell Signaling Technology, #8418. 1:2,000), ZEB1 (Cell Signaling Technology, #3396. 1:2,000), and HA-tag (Cell Signaling Technology, #3724. 1:2,000).

### Cell Migration/Invasion Assays

Control cells and cells with enforced expression of GATA3, Hnf4a, HHEX, or Foxa3 were subjected to migration/invasion assays using the method as described (Xu et al., 2021). Briefly, 4 × 10^5^ U118MG cells, 5 × 10^4^ NE-4C cells, or 2 × 10^5^ HCT116 cells were suspended in 200 μl of serum-free culture medium and added to the upper compartment of a well of a 24-well transwell plate with inserts of 8-μm pore size, and 500 μl of culture medium containing 10% FBS was added in the lower compartment. After incubation of the plates at 37°C for a desired time period as indicated in the text, cells were fixed with 37% formaldehyde and then stained with 0.5% crystal violet for 10 min. After the removal of cells that did not migrate, migrated cells were washed with PBS and photographed.

To perform cell invasion assay, each 80 μl of Matrigel (Corning, #354234) was diluted in eight volumes of PBS, and then distributed uniformly onto a 24-well transwell insert. 4 × 10^5^ U118MG cells, 1 × 10^5^ NE-4C cells, or 1 × 10^6^ HCT116 cells were added to Matrigel. Inserts were put in the culture medium in the lower compartment. Plates were incubated at 37°C for the desired time periods as indicated in the text. Afterward, cells were processed in the same way as in the migration assay.

### Soft Agar Colony Formation Assay

Colony formation assay was performed as described (Xu et al., 2021). The top layer of agar was 0.35% of low melting agarose (BBI, #AB0015), and the bottom layer was 0.7%. Each 2,000 cells were distributed in a well of a six-well culture plate and cultured for the desired time periods, as indicated in text. Experiments were performed in triplicate. Significance of difference in colony formation was calculated using unpaired Student’s *t*-test.

### Total RNA Preparation and Reverse Transcriptase-Quantitative Polymerase Chain Reaction (RT-qPCR)

Reverse transcriptase-quantitative polymerase chain reaction was performed as described (Xu et al., 2021). Total RNAs were extracted from cells using TRIzol following the protocol provided by the manufacturer. cDNAs were reverse transcribed from total RNAs using the HiScript II First Strand cDNA Synthesis Kit (Vazyme, #R212-01/02), which contains reagent for the removal of genomic DNA contamination. qPCR was performed on a LightCycler^®^ 96 system (Roche) using the following parameters: one cycle of pre-denaturation at 95°C for 5 min, followed by 40 cycles of denaturation at 95°C for 10 s, annealing and extension at 60°C for 30 s, and an additional cycle for melting curve. Transcription level of *b-ACT* was detected as a loading control. Significance in gene expression change was calculated based on experiments in triplicate using unpaired Student’s *t*-test. Final results were presented as histograms with relative units of transcription levels. Primers for qPCR are listed in Supplementary Table 1.

### Data Analysis

To analyze genome binding of FOXA3, ChIP-Seq data for FOXA3 in HepG2 were downloaded from the GEO database under the data series GSE104247. The dataset for chromatin fragments immunoprecipitated with antibody was GSM2797524, and the dataset for Input was GSM2797699 (Partridge et al., 2020). Analysis was performed essentially as described (Liu et al., 2020) with modifications. Briefly, single-end reads were mapped to the human genome (UCSC hg38) using Bowtie2 (Version 2.2.5). Only the sequences uniquely mapped with no more than one mismatch were kept and used as valid reads. Replicate bam files of FOXA3 were subsequently merged using Samtools (Version 1.1). Peaks were identified with the peak-calling program MACS2 (Version 2.1.1) using the following parameter settings: -keep-dup = 1, -B, -SPMR. Gene annotation of the peaks was performed by using HOMER (Version 4.11.1). Genes associated with peak centers locating within promoter region −1000 to +100 base from transcription start sites from the *UCSC* genome browser were used for further analysis (Supplementary Table 2).

Meanwhile, differentially expressed genes (DEGs) in HepG2 cells overexpressing FOXA3 and control cells were analyzed using RNA-seq data that were downloaded from the GEO database under data series GSE115423. Dataset for HepG2 cell overexpressing FOXA3 for 7 days was GSM3177984, and the dataset for control HepG2 cells expressing EGFP for 7 days was GSM3177986 (Takashima et al., 2018). Genes with differential expression between cells with FOXA3 overexpression and control cells were calculated. Genes with FPKM value lower than 1 in both control and treatment samples were excluded for calculation. A gene was considered as differentially expressed if | log2FC| ≥ 1. DEGs are listed as Supplementary Table 3.

Differentially expressed genes that are associated with ChIP-seq peaks above were considered as putative target genes of FOXA3 (Supplementary Table 4). A list of 1011 putative target genes of HHEX (Supplementary Table 5) was downloaded from the Molecular Signatures Database (MSigDB) (Subramanian et al., 2005). Function annotation of FOXA3 or HHEX target genes was analyzed using DAVID annotation tools (Huang et al., 2007) with default settings.

### Luciferase Assay

Luciferase reporters for gene promoters were used for detecting the effect of enforced expression of HHEX or Foxa3 on transcription of *SOX2*, *CDKN1A*, and *SBDS*, respectively. HEK293T cells at 80% confluency were co-transfected with 250 ng of luciferase reporter plasmid, 1 ng of Renilla luciferase reporter plasmid (Promega), and 250 ng of HHEX or Foxa3 expression plasmid. As a control, the empty luciferase reporter vector plasmid and the empty vector plasmid for expression constructs were transfected in parallel in the same way. 24 h later, luciferase activity was measured with the Dual-Luciferase Reporter Assay System (Promega, #E1960) according to the manufacturer’s instructions. Results are displayed as relative units of luciferase activity that were obtained from at least three independent experiments. Significance in change of luciferase activity was calculated using unpaired Student’s *t*-test.

### Chromatin Immunoprecipitation (ChIP)

HCT116 cells with enforced expression of HA-tagged HHEX (HA-HHEX) or Foxa3 overexpression were used for ChIP assay, which was performed essentially as described (Lei et al., 2019). In brief, protein–DNA complexes were crosslinked with 1% of formaldehyde solution for 10 min at room temperature. 1/20 volume of 2.5 M glycine was added to terminate crosslinking for 5 min. Cells were then washed thrice with ice-cold PBS, trypsinized, and washed again with PBS. Cells were lysed, and cell nuclei were precipitated. After re-suspension, cell nuclei were lysed for 20 min at 4°C in nuclear lysis buffer (1% SDS, 10 mM EDTA, 50 mM Tris-HCl pH8.0) supplemented with protease inhibitors. ChIP dilution buffer (0.01% SDS, 1% Triton X-100, 2 mM EDTA, and 20 mMTris-HCl pH 8.0), 150 mM NaCl, plus protease inhibitors) was added to nuclear lysates. Afterward, samples were then sonicated with 10 cycles of 30 s pulse followed by 30 s rest with a sonicator (Bioruptor^TM^ USD-200) under high power output. After centrifugation, the supernatants were pre-cleared with protein-G agarose that was pre-blocked with 1% BSA in PBS. 50 μl of pre-cleared chromatin was set aside as input control. The remaining part was divided into equal two parts and incubated with 3 μg of antibody against HA-tag (Cell Signaling Technology, #3724), Foxa3 (Santa Cruz, #sc-74424) or mouse IgG at 4°C for overnight on a rocking platform. Afterward, the immunocomplexes were collected by precipitation with pre-blocked protein-G agarose 4°C for 4 h with gentle rocking. DNA was eluted with reversal of crosslinking and proteinase K digestion, extracted, and precipitated with the conventional phenol-chloroform-ethanol method. Primer pairs flanking the recognition motifs of HHEX or Foxa3 in the promoter regions of *SOX2*, *C-MYC*, *FOXM1*, *EZH2*, *SBDS*, and *AFP* (positive control) were used to detect the binding of a protein with promoters with qPCR, which was performed in the same way as in RT-qPCR. After normalization against the levels of input, changes in protein–DNA binding in the detected promoters were compared by calculating the levels of DNA fragments immunoprecipitated by IgG and by specific antibody (anti-HA-tag or Foxa3). Significance in difference of levels of precipitated chromatin was calculated using unpaired Student’s *t*-test based on experiments in triplicates. Primers are listed in Supplementary Table 1.

### Fluorescence-Activated Cell Sorting

HCT116 cells were infected with lentivirus derived from pLVX-Foxa3-IRES-ZsGreen1 or from the empty vector pLVX-IRES-ZsGreen1, respectively, for 3 days. Cells were trypsinized, washed, and resuspended in PBS containing antibiotics, 1% FBS, and 1 mM EDTA, followed by filtering with a cell strainer of pore size 40 μm (FALCON, #352340). Filtered cells were subjected to cell sorting on a BD LSRFortessa flow cytometer (BD Biosciences) using the green fluorescence channel. Sorted cells were collected and cultured until they grew to a desired number required for xenograft assay. The sorted cells were separated mainly into two parts. One part was injected directly; the other part was mixed with untreated (non-infected) HCT116 cells at the ratio of 2:1, and then injected into nude mice for tumor formation. In addition, a small portion of sorted cells was cultured defined serum-free medium Ndiff 227 (CellArtis, #Y40002) for neurosphere formation.

### Xenograft Assay

Animal use (including AOM/DSS-induced colitis-associated colon cancer model below) in the study was approved by and in accordance with the guidelines of the Institutional Animal Care and Use Committee (IACUC) at the Model Animal Research Center of Nanjing University (Animal Protocol No.: CY05). Five- to 6-week-old athymic Foxn1^nu^ nude mice were purchased from the National Resource Center for Mutant Mice (Nanjing, China) and maintained in a SPF facility. Different numbers of cells were suspended in 100 μl of sterile PBS and injected subcutaneously into the dorsal flank of mouse. Cell types and injected cell numbers are listed in Supplementary Table 6. Tumor volume was measured periodically. Before tumors grew to the size of 1.5 cm in diameter, mice were sacrificed. Tumors were excised and weighed. Significance of difference in tumor weight between two groups of mice was calculated with unpaired Student’s *t*-test. Tumor volume was calculated using the formula: length × width^2^/2. The significance of difference in tumor volumes between two groups was calculated using two-way ANOVA-Bonferroni/Dunn tests. After measurement, tumor tissues were used for total RNA preparation, protein extraction and cryosectioning.

### AOM/DSS-Induced Colitis-Associated Colon Cancer Model

The AOM/DSS-induced colitis-associated colon cancer model was generated as described (Zaki et al., 2010) to evaluate the effect of HHEX/Foxa3 on tumorigenesis *in vivo*. Briefly, 40 male C57BL/6J mice of 7 weeks old were separated randomly into control and treatment groups. Mice were first anesthetized using a mix of ketamine at 100 mg/kg and xylazine at 5 mg/kg via intraperitoneal injection in a biosafety cabinet. The first time of AAV enema was performed with mice in the control group using virus particles (1.6 × 10^11^ vg/mouse) packaged with empty AAV8 vector, and with mice in the treatment group using a mix of virus particles packaged with AAV8-HHEX (0.8 × 10^11^ vg/mouse) and AAV8-Foxa3 (0.8 × 10^11^ vg/mouse) vectors for expressing HHEX and Foxa3 *in vivo*. After enema, mice were kept upside down for 1 min so that virus particles could distribute uniformly in the colon. One week later, all mice were injected with AOM (Sigma-Aldrich, #25843-45-2) intraperitoneally at a dose of 12.5 mg/kg per mouse. Another week later, three cycles of DSS (36–50 kDa, MP Biomedicals, Costa Mesa, CA, United States) treatment were performed by feeding mice with drinking water containing 2.8% DSS. Each cycle lasted 7 days, with a 2-week interval between DSS cycles. After the second DSS cycle, the second AAV enema was performed in the same way as the first time. 12 weeks after AOM injection, mice were sacrificed. Colons were excised, cut longitudinally, and washed with ice-cold PBS. Colon weight and length were measured, and weight-to-length ratio was calculated (Kjellev et al., 2006). Tumor size was measured, and the number of tumors was counted for each mouse. Colon tissues and tumor samples were fixed with 4% PFA and stored at −80°C for the use for subsequent examinations. A few mice were sacrificed 1 week after the second AAV enema for detecting the expression effect of AAV.

Significance in difference of colonic weight-to-length ratio between control and treatment groups was calculated with unpaired Student’s *t*-test. Significance in difference of tumor numbers was calculated with unpaired Student’s *t*-test.

### Cryosectioning

The xenograft tumors or colon tissues were fixed in 4% PFA, dehydrated in 30% sugar, and then embedded in Tissue-Tek OCT (SAKURA, #4583) and frozen with liquid nitrogen. Tissues were sectioned with a cryostat microtome (Leica). The sections were placed at room temperature for 30 min and then washed with PBS. Green fluorescence was observed under a fluorescence microscope (ZEISS LAM880). Cell nuclei were counterstained with DAPI.

### Histology and Immunohistochemistry (IHC)

Sectioning of paraffin-enbedded colon tissue and subsequent hematoxylin and eosin (HE) staining was performed using the conventional method. IHC assay on sections of colon tissues was performed as described (Xu et al., 2021). Briefly, sections were deparaffinized and rehydrated with sequential series of washes in xylene, 100% ethanol, 95% ethanol, and dH_2_O. Antigen retrieval was performed by steaming slides in 0.01 M sodium citrate buffer (pH 6.0). Sections were washed in dH_2_O, followed by incubation in 3% H_2_O_2_ for 30 mins. After washing with PBS, sections were blocked with 5% bovine serum albumin in PBS, and incubated with primary antibodies against c-Myc (Abcam, #ab32072. 1:100) or Pcna (Abclonal, #A0264. 1:400) diluted in blocking buffer overnight at 4°C. Afterward, sections were washed in PBS and incubated with HRP-conjugated goat anti-rabbit secondary antibody (Jakson, #135695. 1:500). Signals were visualized with DAB substrate (BBI, #E670033). Nuclei were counterstained with hematoxylin. IHC signals were quantified with ImageJ^1^. Signal intensity of a cell was graded from 0 to 3 (0, negative; 1, weak; 2, moderate; and 3, strong), and percentage of cells with different grade in a section was calculated. The IHC score of a section was the sum of the product of multiplying different signal intensity grade and their respective percentage of cells (Qu et al., 2018). To calculate the significance in difference of IHC score, normal or tumor tissues of three mice of each group (untreated, AOM/DSS + vector, and AOM/DSS + HHEX/Foxa3), five to eight different IHC images of each normal tissue or tumor were quantified and significance was analyzed using unpaired Student’s *t*-test. IHC score were presented as histograms.

## Results

### HHEX Inhibits Neural Property in the Neuroblastoma Cell Line SH-SY5Y

**FIGURE 1:**
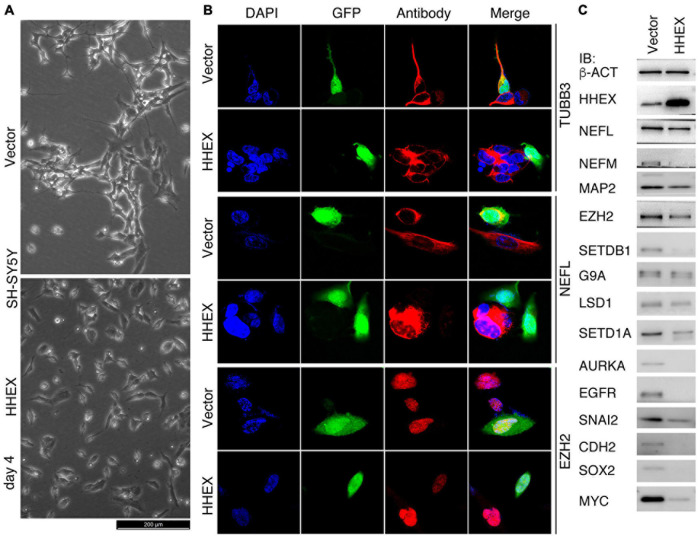
Suppressive effect of HHEX on the neural feature of SH-SY5Y cells. **(A)** Phenotypic alteration of SH-SY5Y cells in response to enforced expression of HHEX after 4 days of virus infection. Cells infected with lentivirus derived from empty vector were used as control (Vector). **(B)** IF detection of expression of neuronal and neural stemness proteins in cells with enforced HHEX expression. Cell nuclei were counterstained with DAPI. **(C)** IB detection of expression of a series of proteins in response to HHEX expression.

SH-SY5Y has been used as a cell model for the study of Parkinson’s disease (Xicoy et al., 2017). We deduced that HHEX should be able to repress the neural property of SH-SY5Y cells. Control cells infected with lentivirus carrying only the vector (Vector) grew neurite outgrowth at day 4 after viral infection, whereas enforced expression of HHEX via lentiviral infection caused repression of neurite outgrowth in the cells, which showed distinct morphology from the control cells (Figure 1A). Immunofluorescence (IF) demonstrated that the neuronal marker TUBB3 and NEFL were detected in control cells but were not detected in cells with enforced expression of HHEX (Figure 1B). EZH2 is an oncoprotein with gene transcription being localized to neural cells during embryogenesis and plays a role in maintaining neural stemness (Zhang et al., 2017; Lei et al., 2019). The protein was detected in control cells, but was strongly downregulated in response to HHEX expression (Figure 1B). Immunoblotting (IB) revealed a strong HHEX overexpression, which caused a downregulation of neuronal proteins NEFL, NEFM, and MAP2, and neural stemness proteins CDH2 and SOX2 (Figure 1C). Repressed proteins also included SNAI2, AURKA, MYC, and EGFR (Figure 1C), which are well-characterized oncoproteins that promote different aspects of cell malignancy or tumorigenicity, such as promotion of cell cycles and migration. Besides EZH2, typical epigenetic modification enzymes and cancer-promoting factors, SETDB1, G9A, and SETD1A, were inhibited (Figure 1C). Gene transcription of these proteins is all enriched in embryonic neural cells during vertebrate embryogenesis (Zhang et al., 2017; Xu et al., 2021). These results reveal that HHEX inhibits neural property in the cells.

### HHEX and Foxa3 Inhibit Malignant Features of U118MG Glioblastoma Cells and Neural Stemness of NE-4C Neural Stem Cells

**FIGURE 2:**
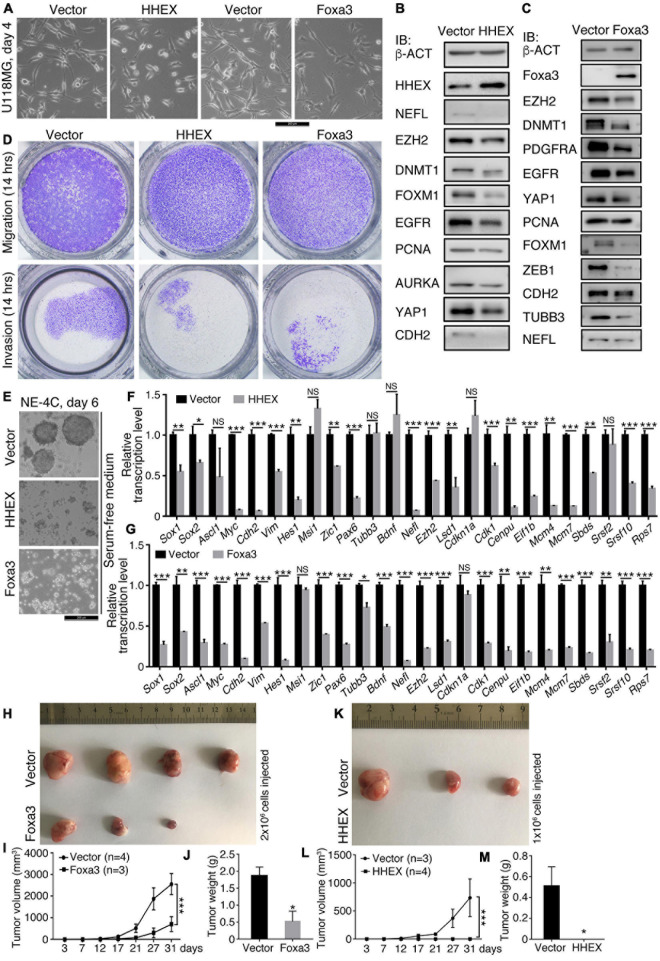
HHEX or Foxa3 suppresses malignant features or tumorigenicity in U118MG and NE-4C cells. **(A–D)** Effect of enforced expression of HHEX or Foxa3 on U118MG cells. **(A)** Phenotypic change in U118MG cells in response to enforced expression of HHEX or Foxa3. IB detection of a series of cancer-promoting factors and neuronal markers in cells with and without (Vector) enforced expression of HHEX **(B)** or Foxa3 **(C)**. IB was performed with whole cell lysate. b-ACT was detected as a loading control. **(D)** Effect of HHEX or Foxa3 expression on the capability of cell migration and invasion. **(E–M)** Effect of enforced expression of HHEX or Foxa3 on NE-4C cells. **(E)** The influence of HHEX or Foxa3 expression on neurosphere formation in NSC specific serum-free medium in the time period as indicated. RT-qPCR detection of changes in transcription of genes representing neural stemness, neuronal differentiation, and genes involved in the basic cellular physiological machineries in control cells (Vector) and cells with HHEX **(F)** or Foxa3 **(G)** expression. Significance in change of transcription level was calculated for experiments in triplicate using unpaired Student’s *t*-test. Data are shown as mean ± SEM. *p < 0.05, **p < 0.01, and ***p < 0.001. NS, not significant. Effect of expression of Foxa3 **(H–J)** or HHEX **(K–M)** on tumorigenicity of NE-4C, as assayed with xenograft tumor formation **(H,K)** in immunodeficient nude mice. Significance of difference in tumor volumes **(I,L)** between two groups of mice was calculated using two-way ANOVA-Bonferroni/Dunn test. Significance of difference in tumor weight **(J,M)** was calculated using unpaired Student’s *t*-test. Data are shown as mean ± SEM. *p < 0.05 and ***p < 0.001.

We examined the effect of HHEX and another non-neural factor, Foxa3, on the neural stemness of glioblastoma cell U118MG and NSC NE-4C. Enforced expression of HHEX or Foxa3 caused significant morphological change in U118MG cells (Figure 2A) and changes in expression of a series of cancer-promoting factors (Figures 2B,C). U118MG also expressed HHEX, whose level was elevated upon overexpression (Figure 2B). The neuronal protein NEFL was detected at a low level, which was inhibited upon HHEX overexpression. The overexpression also caused a decrease in expression of cancer-promoting factors EZH2, DNMT1, FOXM1, EGFR, PCNA, AURKA, YAP1, and CDH2 (Figure 2B). Enforced expression also caused decreased expression of a similar set of cancer-promoting factors EZH2, DNMT1, PDGFRA, EGFR, YAP1, PCNA, FOXM1, ZEB1, and CDH2, and the neuronal proteins TUBB3 and NEFL (Figure 2C). Therefore, HHEX or Foxa3 generated an effect similar to that in SH-SY5Y cells. Enforced HHEX or Foxa3 expression led to repression of cell migration and invasion (Figure 2D), consistent with the repression effect of cancer-promoting factors. In NE-4C cells, HHEX or Foxa3 resulted in the loss of neural stemness because the control cells formed neurospheres in NSC-specific serum-free medium, but the cells with HHEX or Foxa3 expression could not (Figure 2E). They also led to a loss of the capability of migration and invasion in NE-4C cells (Supplementary Figure 1). HHEX caused a repression of a series of neural stemness genes or/and genes promoting cancer, *Sox1*, *Sox2*, *Myc*, *Cdh2*, *Vim*, *Hes1*, *Zic1*, *Pax6*, *Ezh2*, and *Lsd1* (Figure 2F). Interestingly, repressed genes also include those involved in basic cell physiological functions, such as *Cdk1* and *Cenpu* in cell cycle, *Mcm4* and *Mcm7* in DNA replication, *Eif1b* in protein translation, *Sbds* and *Rps7* in ribosome biogenesis, and *Srsf2* and *Srsf10* in RNA splicing. Enforced expression of Foxa3 in NE-4C cells led to a similar tendency of change in transcription of a similar set of genes (Figure 2G). Therefore, HHEX and Foxa3 are on the one hand involved in the stimulation of genes for specifying mesodermal and endodermal tissues or organs during vertebrate embryogenesis, and on the other, repress the neural property in NSCs, which are tumorigenic (Xu et al., 2021). Accordingly, in xenograft assays with immunodeficient nude mice, enforced Foxa3 (Figures 2H–J) or HHEX expression (Figures 2K–M) significantly inhibited tumorigenicity of NE-4C cells (Supplementary Table 6). Expressions of the detected proteins or genes above are all enriched in neural stem cells or embryonic neural cells during embryogenesis (Zhang et al., 2017; Cao, 2020; Xu et al., 2021). This result reinforces that neural stemness is required for cell tumorigenicity, and loss of neural stemness means the reduction or loss of cell tumorigenicity (Xu et al., 2021).

### Inhibitory Effect of Non-neural Pro-differentiation Factors on Other Types of Cancer Cells

**FIGURE 3:**
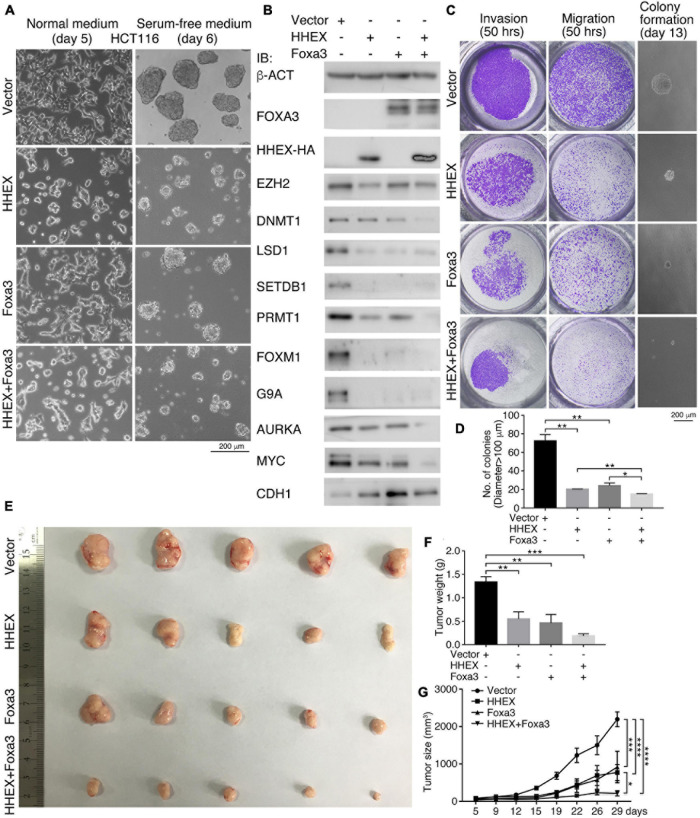
HHEX, Foxa3, or their combination suppresses neural stemness and tumorigenicity in HCT116 cells. **(A)** Morphological alteration in HCT116 cells in normal culture medium and change in ability of neurosphere-like formation in NSC-specific serum-free medium in response to enforced expression of HHEX, Foxa3, or their combination. **(B)** IB detection of protein expression alteration in response to enforced expression of HHEX, Foxa3, and their combination. **(C,D)** Effect of expression HHEX, Foxa3, or their combination on invasion, migration, and colony formation in soft agar **(C)**. Significance in difference of colony formation was calculated based on experiments in triplicate using unpaired Student’s *t*-test **(D)**. Colonies larger than 100 μm in diameter were counted. Data are shown as mean ± SEM. *p < 0.05 and **p < 0.01. **(E–G)** Xenograft tumor formation **(E)** by control HCT116 cells (Vector) and cells with enforced expression of HHEX, Foxa3, and both. Significance of difference in tumor weight between two groups of mice was calculated using unpaired Student’s *t*-test **(F)**, and significance of difference in tumor volume was calculated using two-way ANOVA-Bonferroni/Dunn test **(G)**. Data are shown as mean ± SEM. *p < 0.05, **p < 0.01, ***p < 0.001, and ****p < 0.0001.

In the above, we observed the inhibitory effect of enforced expression of HHEX and Foxa3 on tumor cells derived from the nervous system and NSCs. Next, we asked whether the non-neural pro-differentiation factors exhibit inhibitory effect on other types of tumor cells. First, enforced expression of HHEX or Foxa3 individually or in combination in colon cancer cell line HCT116 caused discernible change in cell morphology, as compared with control cells (Figure 3A). When these cells were cultured in NSC-specific serum-free medium, control cells formed neurosphere-like structures, an indication of neural stemness (Figure 3A). However, cells with enforced expression of HHEX or Foxa3 individually or in combination formed smaller spherical structures (Figure 3A), suggesting an inhibitory effect on neural stemness. Enforced expression of the proteins resulted in repressed expression of factors that promote tumorigenesis, EZH2, LSD1, SETDB1, PRMT1, FOXM1, G9A, AURKA, and MYC (Figure 3B). By contrast, CDH1, which represents epidermal cells during embryogenesis (Zhang et al., 2017) or generally epithelial cells, was upregulated (Figure 3B). Noteworthy is that combined expression of HHEX and Foxa3 achieved a stronger effect on protein expression than expression of a single protein (Figure 3B). The data demonstrate that HHEX or Foxa3 or their combination causes a phenotypic change in HCT116 cells. We observed a significant suppression of invasion, migration, and anchorage-independent growth in soft agar (Figures 3C,D) by HHEX or Foxa3, and double expression led to a stronger repression effect (Figures 3C,D). Xenograft assays showed that enforced expression of either HHEX or Foxa3 inhibited tumorigenicity of HCT116 cells, and combined expression exhibited stronger inhibitory effect (Figures 3E–G and Supplementary Table 6).

**FIGURE 4:**
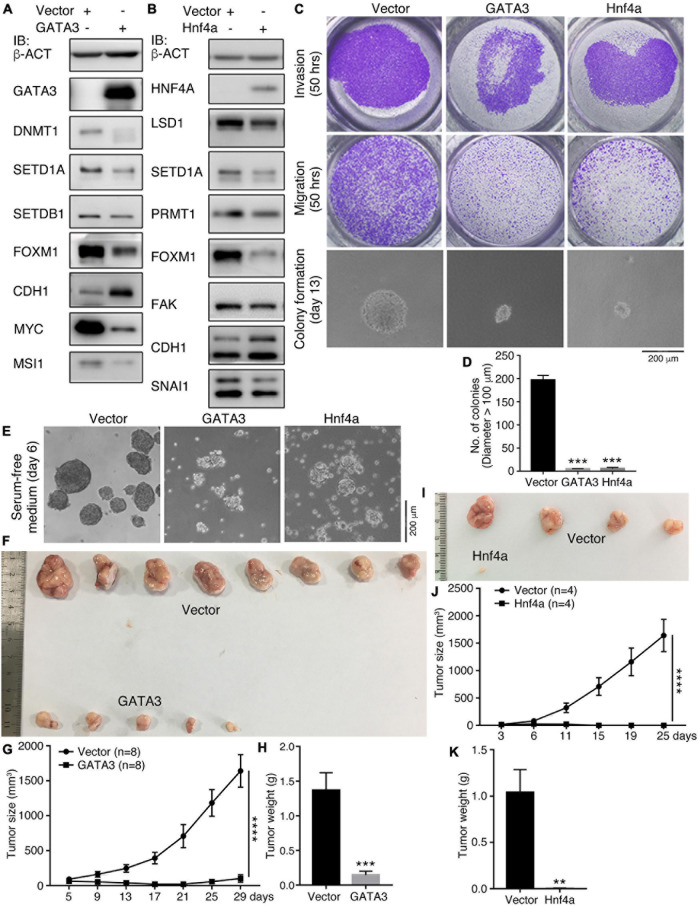
Effect of GATA3 and Hnf4a on malignant features and tumorigenicity of HCT116 cells. **(A,B)** IB detection of protein expression changes in cells in response to enforced GATA3 **(A)** or Hnf4a **(B)** expression. **(C,D)** Detection of invasion, migration, and colony formation of control cells (Vector) and cells with enenforced GATA3 or Hnf4a expression. Significance of difference in colony formation was calculated based on experiments in triplicate using unpaired Student’s *t*-test **(D)**. Data are shown as mean ± SEM. ***p < 0.001. Colonies with a diameter > 100 μm were counted. **(E)** Difference in the ability of neurosphere-like formation of control cells and cells with enenforced GATA3 or Hnf4a expression in NSC-specific serum-free medium. Effect of enenforced GATA3 **(F–H)** or Hnf4a **(I–K)** expression on xenograft tumor formation of HCT116 cells in nude mice. Significance of difference in tumor size **(G,J)** was calculated using two-way ANOVA-Bonferroni/Dunn test, and significance of difference in tumor weight **(H,K)** was calculated using unpaired Student’s *t*-test. Data are shown as mean ± SEM. **p < 0.01, ***p < 0.001, and ****p < 0.0001.

We examined additionally whether GATA3 or Hnf4a affects the malignant features and tumorigenicity of cancer cells. Enforced expression of GATA3 in HCT116 cells inhibited a series of cancer-promoting proteins, DNMT1, SETD1A, SETDB1, FOXM1, MYC, and MSI1 (Figure 4A). Conversely, the epithelial protein CDH1 was upregulated (Figure 4A). Hnf4a expression resulted in a decreased expression of cancer-promoting factors LSD1, SETD1A, PRMT1, FOXM1, FAK, and SNAI1, and also an increased expression of CDH1 (Figure 4B). Expression of these proteins is all enriched in embryonic neural cells or is neural stemness markers except CDH1, which is enriched in epidermis during embryogenesis (Zhang et al., 2017). These data suggest that the GATA3 or HNF4A converts the property of cancer cells to the property of epidermal or epithelial cells. The malignant features, invasion, migration, and colony formation in soft agar (Figures 4C,D), were repressed in response to GATA3 or Hnf4a expression. Moreover, GATA3 or Hnf4a abolished or compromised neurosphere-like formation by HCT116 cells in NSC-specific serum-free medium (Figure 4E), implying a repression effect of neural stemness in cells. Congruent with these effects, tumorigenicity of HCT116 cells was suppressed by either GATA3 (Figures 4F–H and Supplementary Table 6) or Hnf4a (Figures 4I–K and Supplementary Table 6) in xenograft assays. In summary, non-neural pro-differentiation factors exhibit suppression effect on cell tumorigenicity.

### HHEX or Foxa3 Represses Transcription of Genes That Are Enriched in Embryonic Neural Cells

**FIGURE 5:**
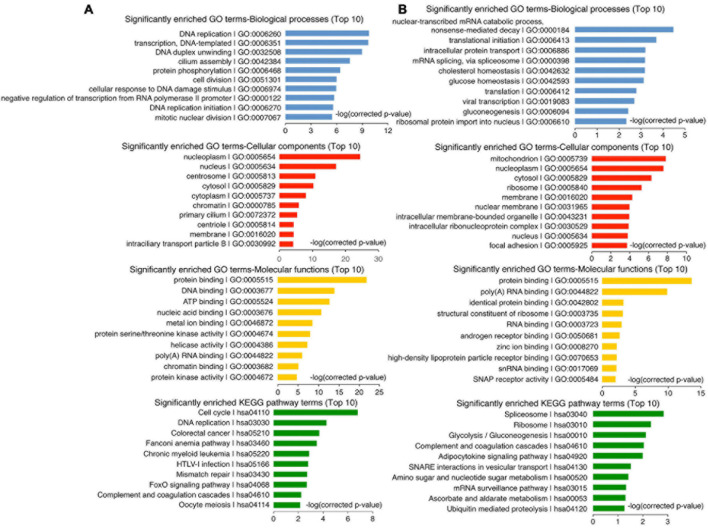
Enrichment analysis on putative target genes of FOXA3 **(A)** and HHEX **(B)**.

**FIGURE 6:**
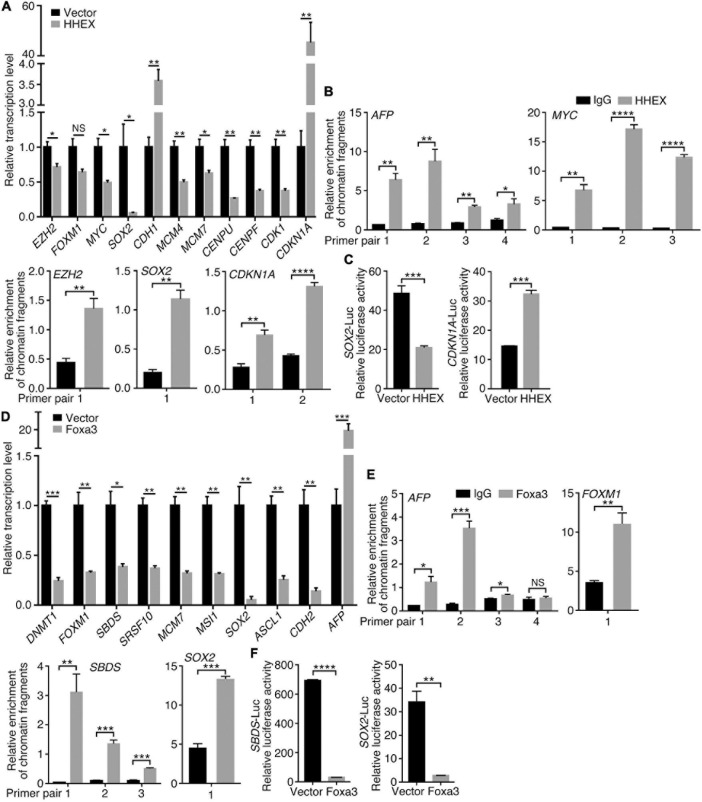
Regulation of target gene trasncription by HHEX or Foxa3. **(A–C)** Differential regulation of gene transcription by enforced expression of HHEX. **(A)** RT-qPCR detection of gene transcription change. **(B)** Binding of HHEX to promoters of different genes as assayed with ChIP followed by qPCR. Different primer pairs denote different regions of a promoter that contain the putative HHEX recognition motifs and were detected with PCR. **(C)** Regulatory effect of HHEX on its target genes that was detected with promoter luciferase reporter assay. **(D–F)** Differential regulation of gene transcription by enforced expression of Foxa3. **(D)** Change in gene transcription detected with RT-qPCR. **(E)** Binding of Foxa3 to different regions of promoters as revealed by ChIP and subsequent qPCR detection. **(F)** Promoter luciferase reporter assay of the regulatory effect of Foxa3 on its target genes. Significance of difference in gene transcription **(A,D)**, DNA-binding **(B,E)**, and luciferase activity **(C,F)** was calculated based on experiments in triplicate using unpaired Student’s *t*-test. Data are shown as mean ± SEM. *p < 0.05, **p < 0.01, ***p < 0.001, and ****p < 0.0001. NS, not significant.

To understand how the non-neural pro-differentiation factors, for example, Foxa3 or HHEX, execute their influences on cell tumorigenicity, we examined their global regulation of gene transcription. By analyzing ChIP-seq and RNA-seq data that are available from databases, 2177 genes were identified as putative target genes of FOXA3 (Supplementary Table 4). These genes are primarily associated with machineries of basic cellular functions, DNA replication, transcription, cell division, and their corresponding cellular components, molecular functions and KEGG pathways, and interestingly, associated with colorectal cancer (Figure 5A). This association is almost the same as the functional annotation of DEGs regulated by FOXA3 (Supplementary Figure 2). The downregulated genes involved in basic cellular physiological functions include, for example, *MCM7* (DNA replication), *CENPF* (cell division), *CCND1* (cell cycle), *SRSF10* (spliceosome assembly), *TOP1* (chromatin remodeling) or *EZH2* (Epigenetic modification), etc. By contrast, liver-specific genes, such as *ALB* and *TTR*, and cell cycle inhibiting genes *CDKN1A* and *CDKN2A* are among the upregulated genes in response to FOXA3 overexpression (Supplementary Tables 3, 4). HHEX was identified to bind to the promoters of 1011 genes (Supplementary Table 5), according to the Molecular Signatures Database (MSigDB) (Subramanian et al., 2005). These genes are also mainly associated with machineries for basic cellular functions, such as mRNA catabolic process, translation, mRNA splicing, metabolism, etc., and associated with respective cellular components, molecular functions, and pathways (Figure 5B). Besides many genes that code for proteins being involved in basic cellular processes, such as *CENPU* (cell cycle), *COPS2* (COP9 signalosome), *DDX55* (RNA metabolism), *EIF1B* (translation initiation), *EXOSC3* (exosome), *PSMA2* (proteasome), *RPS7* (ribosome), *SRSF2* (spliceosome), *KDM3A* (epigenetic modification), *TAF1* (transcription initiation), etc., HHEX target genes also include those that are typically transcribed in embryonic mesodermal or endodermal tissues or organs, such as *A2M*, *AFP*, *GATA4*, *TF*, *TTR*, etc. These analyses suggest that FOXA3 and HHEX regulate not only mesodermal/endodermal genes but also the genes for basic cellular processes, which are usually enriched in embryonic neural cells or NSCs (Xu et al., 2021). We explored further their regulatory effect on the genes that promote cancer in HCT116 cells. Similar to the observed effect in NE-4C cells, enforced expression of HHEX led to repression of *EZH2*, *MYC*, *SOX2*, *MCM4*, *MCM7*, *CENPU*, *CENPF*, and *CDK1*. Meanwhile, an upregulation of *CDH1* and *CDKN1A* was detected (Figure 6A), in agreement with the epithelial-like differentiation effect with decreased proliferation capacity (Figures 3A,B). We also detected the binding of HHEX with the promoters of a few of these regulated genes, which contain HHEX recognition motif 5’-ATTAA-3’ (Crompton et al., 1992). Chromatin immunopreciptation (ChIP) revealed that, besides the binding of HHEX to different regions of *AFP* promoter, HHEX binds to *MYC*, *EZH2*, *SOX2*, and *CDKN1A* promoters (Figure 6B), indicating that these are direct target genes of HHEX. Luciferase reporter assay demonstrated that a 373-bp promoter of *SOX2* containing a HHEX recognition motif (Supplementary Figure 3A) was inhibited (Figure 6C), in agreement with downregulation of *SOX2* transcription in response to HHEX expression. However, HHEX stimulates an 898 bp of promoter region of *CDKN1A* (Figure 6C), which contains two HHEX recognition motifs (Supplementary Figure 3A), reflecting an activation effect on *CDKN1A* transcription. These data also indicate that HHEX acts as both a transcriptional repressor and an activator (Brickman et al., 2000; Gaston et al., 2016).

Enforced expression of Foxa3 downregulated transcription of *DNMT1*, *FOXM1*, *SBDS*, *SRSF10*, *MCM7*, *MSI1*, *SOX2*, *ASCL1*, and *CDH2*, but activated *AFP* in HCT116 cells (Figure 6D). Promoter regions of these genes, for example, *SBDS* and *SOX2*, contain FOXA consensus binding motif 5′-T(A/G)TT(G/T)AC-3′ (Motallebipour et al., 2009; Bochkis et al., 2012; Supplementary Figure 3B). *AFP* promoter also contains multiple consensus binding motifs. Foxa3 bound not only to *AFP* promoter but also to the promoters of *FOXM1*, *SBDS*, and *SOX2* (Figure 6E). Promoter activity of *SBDS* and *SOX2* was inhibited by Foxa3 expression (Figure 6F). Therefore, HHEX or Foxa3 does not only play a role in the specification of non-neural tissues/organs via stimulation of non-neural tissue genes, but also repressed the genes that are usually enriched in embryonic neural cells, i.e., the genes that define neural stemness and the ground state of cell tumorigenicity (Xu et al., 2021).

### Tumorigenic Cells With Enforced Expression of Foxa3 and HHEX Are Not Tumorigenic

**FIGURE 7:**
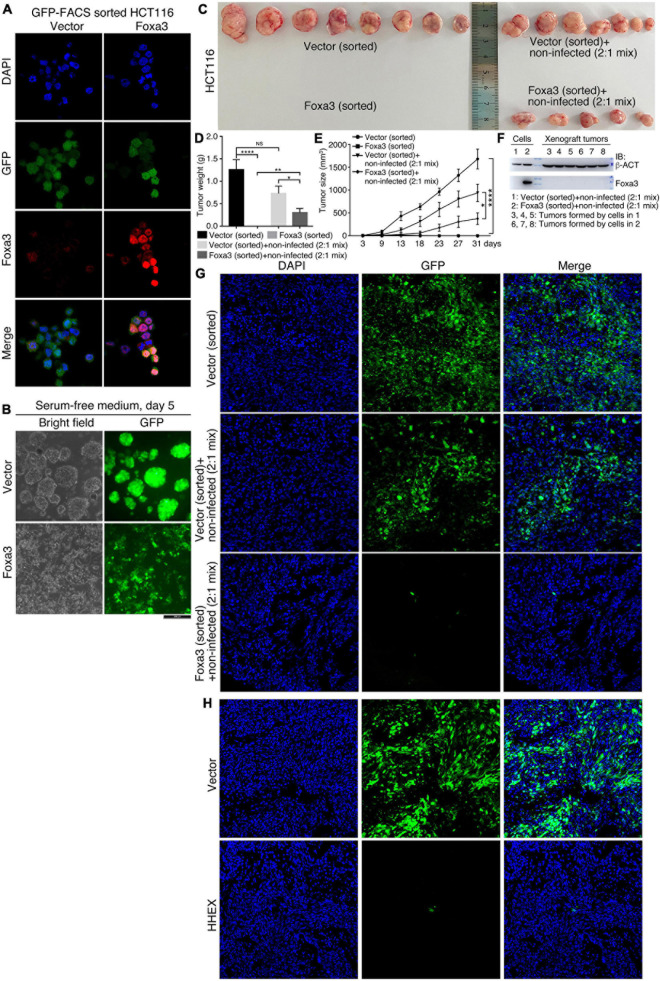
No contribution of cells with enforced Foxa3 or HHEX expression to xenograft tumor formation. **(A)** Examination of Foxa3 expression in HCT116 cells after FACS sorting using GFP. **(B)** The effect of Foxa3 expression on neurosphere-like formation of HCT116 cells after FACS sorting in NSC-specific serum-free medium. **(C)** Xenograft tumor formation by HCT116 cells infected with virus derived from empty vector (Vector) or with virus derived from Foxa3 expression vector followed by FACS sorting, and xenograft tumor formation by a mix of sorted cells and non-infected cells. Significance of difference in tumor weight **(D)** and volume **(E)** between different groups of tumors in **(C)**. Significance of difference in tumor weight was calculated using unpaired Student’s *t*-test, and difference in tumor volume was calculated using two-way ANOVA-Bonferroni/Dunn test. Data are shown as mean ± SEM. *p < 0.05, **p < 0.01, and ****p < 0.0001. NS, not significant. **(F)** IB detection of Foxa3 expression in infected cells and in xenograft tumors formed by cell mixtures, as indicated. **(G)** Cryosections show cells with GFP expression in xenograft tumors derived from injected cells as indicated. **(H)** Cryosections show cells with GFP expression in xenograft tumors derived from HCT116 cells infected with virus carrying the empty vector (Vector) and cells infected with virus carrying HHEX expression vector (HHEX). Cells were not FACS sorted before transplantation into nude mice.

Although HHEX and Foxa3 inhibit neural stemness property and tumorigenicity in both NSCs and HCT116 cells, the cells with enforced expression of these proteins still display the ability to form smaller spherical structures by HCT116 cells and xenograft tumors. We were curious whether this was due to an imperfect selection of lentiviral infection by puromycin, which means that antibiotic selection might not eliminate all the cells with weak or no infection. To answer the question, we expressed Foxa3 in HCT116 cells using a lentiviral vector containing GFP. Cells infected with virus containing the empty vector were used as a control. Before transplantation into nude mice, cells were sorted with FACS using the channel of GFP. IF displayed a high efficiency of cell sorting and Foxa3 expression (Figure 7A). When cultured in NSC-specific serum-free medium, control cells formed neurosphere-like structures, whereas cells with Foxa3 expression did not form any spherical structures and remained attached to the bottom of petri dish. GFP could be detected uniformly in both control cells and cells with enforced expression of Foxa3 (Figure 7B). Injection of sorted control cells led to tumor formation. However, the sorted cells with Foxa3 expression did not form tumor (Figures 7C–E and Supplementary Table 6), an effect somewhat different from injection of antibiotic selected cells. Then we mixed sorted cells, which were infected with virus containing either empty vector or Foxa3, with cells that were not infected (non-infected) with a ratio of 2:1. Both of these mixed cells formed xenograft tumors (Figure 7C and Supplementary Table 6), but the size and weight of tumors derived from the mix of Foxa3-expressing cells and non-infected cells were smaller (Figures 7D,E). Detection of Foxa3 revealed that it was not present in cell mix of vector-infected and non-infected cells, but present in cell mix of Foxa3-infected and non-infected cells. However, Foxa3 could be detected in xenograft tumors derived from both cell mixtures (Figure 7F), suggesting that Foxa3-expressing cells did not contribute to tumor formation. Cryosections demonstrate that tumors derived from the sorted cells infected with vector were predominantly composed of cells expressing GFP, tumors derived from cell mix of vector-infected, and non-infected cells were partially composed of cells expressing GFP. Nevertheless, almost no green fluorescence could be observed from the sections of tumors derived from cell mix of Foxa3-infected and non-infected cells (Figure 7G).

We also detected the contribution of HHEX-expressing cells to xenograft tumor formation. Cells infected with virus containing empty vector or with virus containing HHEX, without FACS sorting, were injected into nude mice for tumor formation (Supplementary Table 6). Cryosections showed that xenograft tumors formed by cells infected with vector contained dominantly GFP-expressing cells, whereas the tumors formed by cells infected with HHEX contained almost no GFP-expressing cells (Figure 7H). These data imply that enforced expression of Foxa3 or HHEX causes the loss of tumorigenicity of HCT116 cells. Xenograft tumor formation by antibiotic selected cells might compromise the repression effect of Foxa3 or HHEX on tumorigenicity.

### Repression Effect of Combined Expression of HHEX and Foxa3 on Tumorigenesis in the AOM/DSS Model of Colitis-Associated Cancer

**FIGURE 8:**
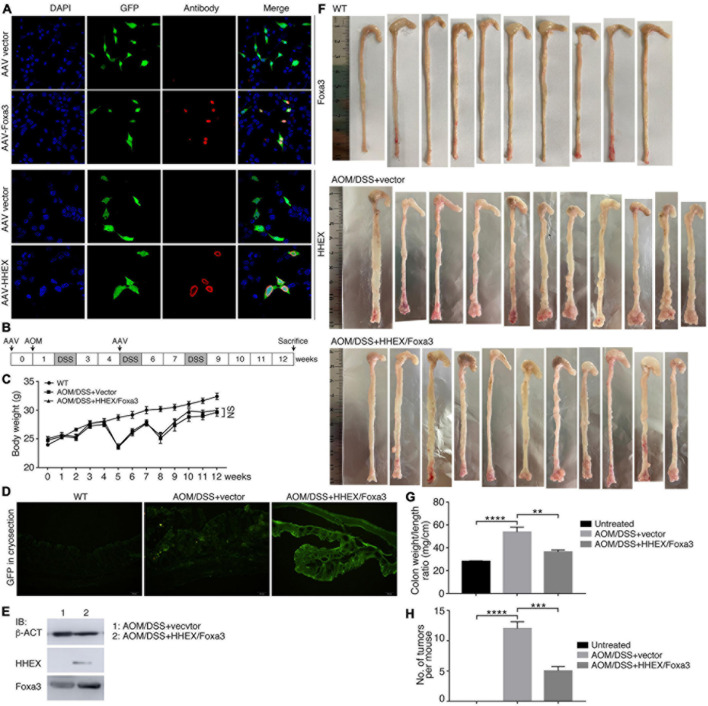
Detection of expression effect of HHEX and Foxa3 in cells and colon tissues, and the effect on colon tumor formation in AOM/DSS model of colitis-associated cancer. **(A)** Detection of expression effect of AAV-HHEX and AAV-Foxa3 constructs in HEK293T cells. Nuclei were counterstained with DAPI. **(B)** Schematic illustration of the strategy for generation of AOM/DSS model of coloretal cancer and time points for AAV administration. **(C)** Body weight change of the wild type mice and mice with AOM/DSS treatment. Significance in body weight between two groups was calculated using two-way ANOVA-Bonferroni/Dunn test. Data are shown as mean ± SEM. NS, not significant. **(D)** Detection of expression effect of the infected AAV virus in colon tissue in different groups via observing GFP in cryosections. **(E)** IB detection of HHEX and Foxa3 expression effect in colon tissues. **(F)** Colons harvested from three groups of mice, as indicated. **(G)** Colonic weight/length ratio in three groups of mice as indicated. **(H)** Numbers of tumors observed in three groups of mice as indicated. In **(G,H)**, significance was calculated using unpaired Student’s *t*-test. Data are shown as mean ± SEM. **p < 0.01, ***p < 0.001, and ****p < 0.0001.

Next, we tested whether HHEX and Foxa3 could repress tumorigenesis in the Azoxymethane/Dextran Sodium Sulfate (AOM/DSS) mouse model of inflammatory colorectal cancer, a well-established and frequently used cancer model. This model also facilitates an effective overexpression of proteins using adeno-associated virus (AAV). Moreover, it has been not reported whether HHEX or Foxa3 or their combination can repress tumorigenesis in colon using a mouse cancer model. We used the AAV vector serotype 8 (AAV8), which is effective for gene transfer and long-term transgene expression in intestine and colon (Polyak et al., 2012), to deliver the genes for HHEX and Foxa3 for expression in colon. The viral vectors could efficiently express HHEX or Foxa3 when transfected in HEK293T cells (Figure 8A). We made the colon cancer model using an established strategy, AAV particles (either control virus or a 1:1 mix of virus for HHEX and Foxa3 expression) were administered twice via enema as indicated (Figure 8B). While the body weight of the untreated group increased constantly, the body weight of mice with chemical treatment fluctuated due to weight loss following repeat cycles of DSS treatment (Figure 8C), an effect that occurs in AOM/DSS model (Parang et al., 2016). Cryosections of colons from mice without AOM/DSS treatment and viral injection (untreated group) showed no green fluorescence. Colon sections from mice with AOM/DSS treatment and injected with virus containing empty vectors (control group) or injected simultaneously with both virus containing HHEX and Foxa3 (protein expression group) displayed significant green fluorescence (Figure 8D), suggesting a successful delivery of the virus and expression of the genes. This was confirmed by detection of protein expression of HHEX and Foxa3. HHEX was not detected in the colon tissues in the control group, but detected in the tissues from the protein expression group (Figure 8E). Foxa3 was detected at a medium level in the tissues from the control group, showing a background expression of the protein in intestinal tissues. In the protein expression group, a much higher level of Foxa3 was detected (Figure 8E).

**FIGURE 9:**
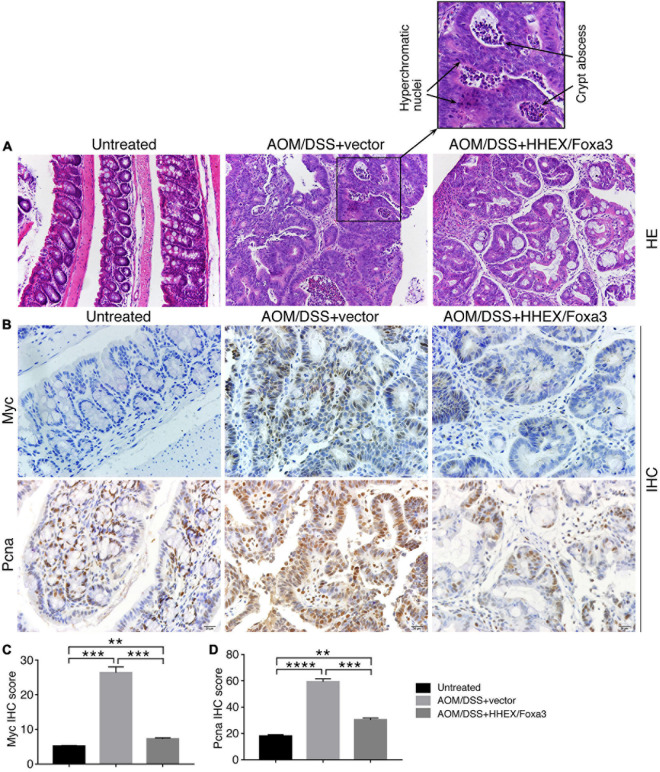
Histological and IHC analysis of the effect of combined expression of HHEX and Foxa3 on tumorigenesis in AOM/DSS model of colitis-associated cancer. **(A)** HE staining of colon tissue from mice of different groups as indicated. The left panel shows normal histological structure of colon tissue. The middle panel shows the section across a tumor, with the enlarged area showing hyperchromatic nuclei and crypt abscess that are usually present in AOM/DSS-induced colon cancer. The right panel shows the section across the tumor in the mice with HHEX/Foxa3 expression, showing less severe pathological characteristics. Objective magnification: 20×. **(B)** IHC detection of Myc and Pcna expression in sections of the tissues from different groups of mice, as indicated. Objective magnification: 20×. Quantification of IHC signals for Myc **(C)** and Pcna **(D)** in sections as in **(B)**. Significance of difference in IHC signal score was calculated using unpaired Student’s *t*-test. Data are shown as mean ± SEM. **p < 0.01, ***p < 0.001, and ****p < 0.0001.

No tumors were observed in colons of the untreated group. By contrast, tumor burdens were present in the colons of AOM/DSS-treated mice, either the control group or the protein expression group, and the tumors were primarily located in the distal colon regions (Figure 8F). Colonic weight-to-length ratio was considered as an indication of clinical and histologiocal severity of colonic disease (Kjellev et al., 2006). The ratio of control group was significantly higher than those of both the untreated and protein expression groups (Figure 8G). The mean number of tumors per mouse in the control group was also significantly higher than other two groups (Figure 8H). Therefore, mice in the control group developed heavier tumor burdens than the mice with expression of HHEX and Foxa3, or HHEX and Foxa3 expression in colon generated a suppressive effect on tumorigenesis. The colon tissues from the untreated mice revealed normal histological structures, whereas the tissues from the control mice exhibited typical pathological features, such as crypt abscesses and hyperchromatic nuclei (Figure 9A). Although tissues from the mice in protein expression group were also abnormal, the pathological features appeared less severe than in the control group, because of the absence of a large crypt abscesses and hyperchromatic nuclei (Figure 9A). MYC is overexpressed and involved in colitis-associated colorectal cancer (Sipos et al., 2016); PCNA is also upregulated in colorectal cancer (Peng et al., 2019). Accordingly, Myc was not detected in the untreated colon samples, but present in a high level in tumors from the control group. A low level of Myc expression was also detected in the samples from the protein expression group (Figure 9B). Pcna was detected in samples from all three groups. However, the expression level in the control group was seemingly higher than that in other two groups (Figure 9B). The difference was also confirmed by quantification of the immunohistochemical signals (Figures 9C,D). In summary, combined expression of HHEX and Foxa3 exhibited a suppressive effect on tumorigenesis in the colitis-associated colorectal cancer model.

## Discussion

Germ layer formation during early embryogenesis and later on tissue differentiation and organogenesis all occur in an exact tempo-spatial pattern, such that a normal animal could develop. This means that genes specify a tissue or an organ must in the meantime repress the genes specifying adjacent or other tissues or organs, thereby maintaining the integrity of the tissue or organ, and establishing the boundaries between different tissues or organs. As we have elucidated that tumorigenic cells have the property of neural stemness, which defines the tumorigenic and differentiation potentials in a cell because of the evolutionary advantage of neural stemness (Zhang et al., 2017; Cao, 2020; Xu et al., 2021). In other words, all embryonic or somatic cell types are built on the ground of neural stemness. During germ layer formation, neural precursor cells are derived from the ectoderm via the double inhibition mechanism, i.e., inhibition of the signals that inhibit neural fate, particularly TGFbeta signaling. The ectodermal cells can be further induced to differentiate into non-neural cells, i.e., the effect of pluripotency. With the progression of differentiation, the neural ground state, and thus the cell tumorigenicity and differentiation potential, is progressively decreased. Therefore, non-neural pro-differentiation factors or signals are usually tumor suppressors, as we generalized previously (Zhang et al., 2017). TGFbeta signaling, which antagonizes neural fate and promotes non-neural differentiation in embryonic pluripotent cells, is characterized as a suppressor of tumor formation. Accordingly, removal of TGFbeta signaling from ESCs generates primitive NSCs, which exhibits stronger tumorigenicity than ESCs (Xu et al., 2021). The present study also emphasizes the inhibitory effect on cell tumorigenicity through repression of neural stemness by non-neural pro-differentiation factors. The transcription factors under current investigation, HHEX, FOXA3, GATA3, and HNF4A, play essential roles in non-neural tissue or organ development. HHEX is usually inactivated or expressed at a low level in different types of cancer cells, including liver, breast, and thyroid cancer, and was shown to inhibit breast tumor growth (Gaston et al., 2016; Kershaw et al., 2017). FOXA3 expression in cancers has not been clear. However, FOXA3 was demonstrated to repress stemness of colorectal cancer cells via targeting MACC1 (Li N. et al., 2020), whose gene expression is enriched in neural cells during early embryogenesis (Melvin et al., 2013). Foxa3 can promote anterior neural tissue formation during zebrafish embryogenesis. However, this effect is achieved indirectly by that Foxa3 protects presumptive anterior neural cells from caudalization by the posteriorizing factor Wnt8a (Seiliez et al., 2006). Moreover, enforced expression of Foxa3, together with other factors, promotes differentiation of ESCs into a hepatic lineage, and converts fibroblasts and even hepatoma cells into hepatocytes or hepatocyte-like cell (Huang et al., 2011, 2014; Yahoo et al., 2016; Cheng et al., 2019). These results mean that Foxa3 does not have an intrinsic neural inducing activity *per se* because it is not expressed in embryonic neural cells but expressed in endodermal tissues during embryogenesis (Monaghan et al., 1993), supporting an intrinsic inducing activity of Foxa3 for endodermal cells, notably hepatic cells. HNF4A as a tumor suppressor in liver and colon cancers, its gene is silenced in the squamous subtype of pancreatic ductal adenocarcinoma (Walesky and Apte, 2015; Brunton et al., 2020). Functional inhibition of HNF4A by a mutant IDH causes a failure in hepatocyte differentiation and an elevated cell proliferation and promotes biliary cancer (Saha et al., 2014). Occasionally, HNF4A was found to be upregulated in gastrointestinal adenocarcinomas and promote the cancer as shown by knockdown assays (Pan et al., 2020). However, no overexpression assay on HNF4A was performed to mimic its elevated expression in cancer cells. Tumors are characteristic of phenotypic heterogeneity due to the differentiation potential of tumorigenic cells (Xu et al., 2021), thereby leading to the generation of different cell types or cells with expression of specific markers. For example, cells with expression of smooth muscle protein ACTA2 are present in many tumors. AFP is not only a biomarker for liver, testis, and ovary cancer, but is also detected in other cancer types including colorectal and gastric cancers and in xenograft tumors of neural stem cells and different cancer cell lines (Yachida et al., 2003; Anzai et al., 2015; Gong et al., 2018; Xu et al., 2021). Their high expression in some tumor cells does not necessarily mean that they promote cancer. It has been debatable whether GATA3, which is extensively investigated in breast cancer, functions to suppress or promote cancer. Mutation of GATA3 is frequently observed in breast cancer, and its expression is lost in mouse models of breast cancer and in upper tract urothelial carcinoma (Takaku et al., 2015; Wang et al., 2019). Similar to our result, enforced GATA3 expression led to increased differentiation and decreased dissemination and metastasis (Kouros-Mehr et al., 2008). In contrast, GATA3 enhances invasiveness of cells of head and neck squamous cell carcinoma and cancer cell stemness in ovarian high-grade serous carcinoma (Lin et al., 2017; Chen et al., 2018). GATA3 is expressed in non-neural cells in early embryogenesis, but it shows enriched expression in neural cells in later embryos (Thisse et al., 2001). The expression of GATA3 during normal embryogenesis suggests that it is involved in the regulation of both non-neural and neural cells, depending on cell context. This manner of function is also reflected by that GATA3 is lost in and suppresses some cancers but promotes some others. Besides the present study, other non-neural pro-differentiation factors exhibit tumor suppressor activity, as we generalized previously (Zhang et al., 2017). For example, *Nkx3-1* is localized to mesodermal tissues during prenatal mouse embryogenesis and contributes to axial skeleton and prostate formation (Tanaka et al., 1999; Herbrand et al., 2002; Abate-Shen et al., 2008). Accordingly, NKX3-1 expression is commonly decreased during tumorigenesis in prostate and functions as a prostatic tumor suppressor (Bowen et al., 2000; Abdulkadir, 2005; Abate-Shen et al., 2008). The tumor suppression effect is achieved partially by NKX3-1 repression of transcription of *TWIST1* (Eide et al., 2013), a gene with localized transcription in neural crest cells during vertebrate embryogenesis (LaBonne and Bronner-Fraser, 1998). Although the functions of these pro-differentiation factors and sometime the underlying molecular mechanisms during tumorigenesis are investigated, they have been focused on specific types of cancers. The mechanisms are mostly about the regulatory relationship between these factors and other individual cancer-related genes or factors. Whether they have a general tumor suppression effect has been unknown. The current study shows that they repress neural stemness (or neural ground state) and promote non-neural properties, suggesting a more extensive tumor suppression effect. Repression of neural stemness by these factors is achieved by binding to genes whose expression is enriched in embryonic neural cells, repressing transcription of neural enriched genes. Meanwhile, they activate genes that confer the property of non-neural cells, for example, the activation of liver-specific genes by FOXA3 (Horisawa et al., 2020) and activation of epithelial gene *CDH1* by HHEX in the present study. Functioning as both a transcriptional repressor and an activator might be a general feature of pro-differentiation transcription factors (Abate-Shen et al., 2008; Gaston et al., 2016).

Based on the analysis of teratocarcinoma, it was proposed that cancer could be considered as developmental disorder (Rubin, 1985). Indeed, all tumors are either teratomas/teratocarcinomas or degenerated or more severely defected forms of teratomas/teratocarcinomas (Xu et al., 2021). Basic principles governing normal embryonic tissue differentiation should also apply to tumor formation. Besides neuronal differentiation in their native environment, NSCs can be induced to differentiate into different cell types with reduced or loss of tumorigenicity. This might be an effective strategy to suppress cancer. Former studies demonstrated that liver cancer cells could be turned into hepatocyte-like cells by a liver specification factor Hnf4a, pancreatic cancer cells could be turned into exocrine cells by a pancreas specification factor Ptf1a, and metastatic breast cancer cells could be turned into adipocytes. These conversions repressed tumorigenesis or metastasis (Yin et al., 2008; Ishay-Ronen et al., 2019; Krah et al., 2019), in congruent with the notion that non-neural pro-differentiation factors inhibit tumorigenicity. Targeted therapy has been the mainstream strategy for cancer therapy. The effect is usually compromised, for example, by complex feedback loops in the regulatory network of cancer cells. Moreover, tumor is of enormous genetic and phenotypic heterogeneity. In most cases, a target is not uniformly present in all tumor cells. Cells without the target, which are possibly also tumorigenic, will escape the targeting. By contrast, pro-differentiation factors or their combination have the ability to reprogram the whole regulatory network, via repression of the neural ground state and meanwhile promotion of non-neural cell property in cancer cells. We suggest that it might be more crucial to target the stem of cancer cells, i.e., neural stemness or neural ground state, in cancer therapy (Cao, 2017; present study).

## Data Availability Statement

The original contributions presented in the study are included in the article/Supplementary Material, further inquiries can be directed to the corresponding author.

## Ethics Statement

The animal study was reviewed and approved by the Institutional Animal Care and Use Committee (IACUC) at the Model Animal Research Center of Nanjing University.

## Author Contributions

YC conceived the research. XY and NC performed cell and animal experiments. XY, NC, and LC performed biochemical experiments. XY, NC, and MZ performed molecular experiments. LL and XY performed bioinformatic analysis. YC wrote the manuscript. All authors analyzed the data.

## Conflict of Interest

The authors declare that the research was conducted in the absence of any commercial or financial relationships that could be construed as a potential conflict of interest.

## Publisher’s Note

All claims expressed in this article are solely those of the authors and do not necessarily represent those of their affiliated organizations, or those of the publisher, the editors and the reviewers. Any product that may be evaluated in this article, or claim that may be made by its manufacturer, is not guaranteed or endorsed by the publisher.

## Footnotes

1
https://imagej.nih.gov/ij/

## References

[B1] Abate-Shen C. Shen M. M. Gelmann E. (2008). Integrating differentiation and cancer: the Nkx3.1 homeobox gene in prostate organogenesis and carcinogenesis. *Differentiation* 76 717–727. 10.1111/j.1432-0436.2008.00292.x 18557759 PMC3683569

[B2] Abdulkadir S. A. (2005). Mechanisms of prostate tumorigenesis: roles for transcription factors Nkx3.1 and Egr1. *Ann. N. Y. Acad. Sci.* 1059 33–40. 10.1196/annals.1339.018 16382041

[B3] Anzai H. Kazama S. Kiyomatsu T. Nishikawa T. Tanaka T. Tanaka J. (2015). Alpha-fetoprotein-producing early rectal carcinoma: a rare case report and review. *World J. Surg. Oncol.* 13:180. 10.1186/s12957-015-0590-x 25962419 PMC4440317

[B4] Bochkis I. M. Schug J. Ye D. Z. Kurinna S. Stratton S. A. Barton M. C. (2012). Genome-wide location analysis reveals distinct transcriptional circuitry by paralogous regulators Foxa1 and Foxa2. *PLoS Genet.* 8:e1002770. 10.1371/journal.pgen.1002770 22737085 PMC3380847

[B5] Bowen C. Bubendorf L. Voeller H. J. Slack R. Willi N. Sauter G. (2000). Loss of NKX3.1 expression in human prostate cancers correlates with tumor progression. *Cancer Res.* 60 6111–6115. 11085535

[B6] Brickman J. M. Jones C. M. Clements M. Smith J. C. Beddington R. S. (2000). Hex is a transcriptional repressor that contributes to anterior identity and suppresses Spemann organiser function. *Development* 127 2303–2315. 10804173 10.1242/dev.127.11.2303

[B7] Brunton H. Caligiuri G. Cunningham R. Upstill-Goddard R. Bailey U. M. Garner I. M. (2020). HNF4A and GATA6 loss reveals therapeutically actionable subtypes in pancreatic cancer. *Cell Rep.* 31:107625. 10.1016/j.celrep.2020.107625 32402285 PMC9511995

[B8] Cao Y. (2017). Tumorigenesis as a process of gradual loss of original cell identity and gain of properties of neural precursor/progenitor cells. *Cell Biosci.* 7:61. 10.1186/s13578-017-0188-9 29177029 PMC5693707

[B9] Cao Y. (2020). Neural is fundamental: neural stemness as the ground state of cell tumorigenicity and differentiation potential. *Preprints* 2020, 2020120122. 10.20944/preprints202012.0122.v1 34714532

[B10] Chen H. J. Huang R. L. Liew P. L. Su P. H. Chen L. Y. Weng Y. C. (2018). GATA3 as a master regulator and therapeutic target in ovarian high-grade serous carcinoma stem cells. *Int. J. Cancer* 143 3106–3119. 10.1002/ijc.31750 30006927

[B11] Cheng Z. He Z. Cai Y. Zhang C. Fu G. Li H. (2019). Conversion of hepatoma cells to hepatocyte-like cells by defined hepatocyte nuclear factors. *Cell Res.* 29 124–135. 10.1038/s41422-018-0111-x 30560924 PMC6355772

[B12] Crompton M. R. Bartlett T. J. MacGregor A. D. Manfioletti G. Buratti E. Giancotti V. (1992). Identification of a novel vertebrate homeobox gene expressed in haematopoietic cells. *Nucleic Acids Res.* 20 5661–5667. 10.1093/nar/20.21.5661 1360645 PMC334400

[B13] Eide T. Ramberg H. Glackin C. Tindall D. Taskén K. A. (2013). TWIST1, A novel androgen-regulated gene, is a target for NKX3-1 in prostate cancer cells. *Cancer Cell Int.* 13:4. 10.1186/1475-2867-13-4 23368843 PMC3626792

[B14] Foley A. C. Mercola M. (2005). Heart induction by Wnt antagonists depends on the homeodomain transcription factor Hex. *Genes Dev.* 19 387–396. 10.1101/gad.1279405 15687261 PMC546516

[B15] Gaston K. Tsitsilianos M. A. Wadey K. Jayaraman P. S. (2016). Misregulation of the proline rich homeodomain (PRH/HHEX) protein in cancer cells and its consequences for tumour growth and invasion. *Cell Biosci.* 13;6:12. 10.1186/s13578-016-0077-7 26877867 PMC4752775

[B16] Gong W. Su Y. Liu A. Liu J. Sun D. Jiang T. (2018). Clinical characteristics and treatments of patients with alpha-fetoprotein producing gastric carcinoma. *Neoplasma* 65 326–330. 10.4149/neo_2018_170207N84 29788728

[B17] Herbrand H. Pabst O. Hill R. Arnold H. H. (2002). Transcription factors Nkx3.1 and Nkx3.2 (Bapx1) play an overlapping role in sclerotomal development of the mouse. *Mech Dev.* 117 217–224. 10.1016/s0925-4773(02)00207-1 12204261

[B18] Horisawa K. Udono M. Ueno K. Ohkawa Y. Nagasaki M. Sekiya S. (2020). The dynamics of transcriptional activation by hepatic reprogramming factors. *Mol. Cell* 79 660–676.e8. 10.1016/j.molcel.2020.07.012 32755593

[B19] Huang D. W. Sherman B. T. Tan Q. Kir J. Liu D. Bryant D. (2007). DAVID Bioinformatics Resources: expanded annotation database and novel algorithms to better extract biology from large gene lists. *Nucleic Acids Res* 35 W169–W175. 10.1093/nar/gkm415 17576678 PMC1933169

[B20] Huang P. He Z. Ji S. Sun H. Xiang D. Liu C. (2011). Induction of functional hepatocyte-like cells from mouse fibroblasts by defined factors. *Nature* 475 386–389. 10.1038/nature10116 21562492

[B21] Huang P. Zhang L. Gao Y. He Z. Yao D. Wu Z. (2014). Direct reprogramming of human fibroblasts to functional and expandable hepatocytes. *Cell Stem Cell* 14 370–384. 10.1016/j.stem.2014.01.003 24582927

[B22] Ishay-Ronen D. Diepenbruck M. Kalathur R. Sugiyama N. Tiede S. Ivanek R. (2019). Gain fat-lose metastasis: converting invasive breast cancer cells into adipocytes inhibits cancer metastasis. *Cancer Cell* 35 17–32.e6. 10.1016/j.ccell.2018.12.002 30645973

[B23] Kershaw R. M. Roberts D. Wragg J. Shaaban A. M. Humphreys E. Halsall J. (2017). Proline-rich homeodomain protein (PRH/HHEX) is a suppressor of breast tumour growth. *Oncogenesis* 6:e346. 10.1038/oncsis.2017.42 28604763 PMC5519192

[B24] Kjellev S. Lundsgaard D. Poulsen S. S. Markholst H. (2006). Reconstitution of Scid mice with CD4+CD25- T cells leads to rapid colitis: an improved model for pharmacologic testing. *Int. Immunopharmacol.* 6 1341–1354. 10.1016/j.intimp.2006.04.017 16782548

[B25] Kouros-Mehr H. Bechis S. K. Slorach E. M. Littlepage L. E. Egeblad M. Ewald A. J. (2008). GATA-3 links tumor differentiation and dissemination in a luminal breast cancer model. *Cancer Cell* 13 141–152. 10.1016/j.ccr.2008.01.011 18242514 PMC2262951

[B26] Krah N. M. Narayanan S. M. Yugawa D. E. Straley J. A. Wright C. MacDonald R. J. (2019). Prevention and reversion of pancreatic tumorigenesis through a differentiation-based mechanism. *Dev. Cell* 50 744–754.e4. 10.1016/j.devcel.2019.07.012 31422917 PMC6776997

[B27] LaBonne C. Bronner-Fraser M. (1998). Neural crest induction in Xenopus: evidence for a two-signal model. *Development* 125 2403–2414. 9609823 10.1242/dev.125.13.2403

[B28] Lei A. Chen L. Zhang M. Yang X. Xu L. Cao N. (2019). EZH2 regulates protein stability via recruiting USP7 to mediate neuronal gene expression in cancer cells. *Front. Genet.* 10:422. 10.3389/fgene.2019.00422 31130994 PMC6510286

[B29] Li N. Li Y. Gao H. Li J. Ma X. Liu X. (2020). Forkhead-box A3 (FOXA3) represses cancer stemness and partially potentiates chemosensitivity by targeting metastasis-associated in colon cancer 1 (MACC1) signaling pathway in colorectal cancer cells. *Curr. Cancer Drug Targets* 10.2174/1568009620666201207150632 [Epub ahead of print]. 33292133

[B30] Li Y. Rankin S. A. Sinner D. Kenny A. P. Krieg P. A. Zorn A. M. (2008). Sfrp5 coordinates foregut specification and morphogenesis by antagonizing both canonical and noncanonical Wnt11 signaling. *Genes Dev.* 22 3050–3063. 10.1101/gad.1687308 18981481 PMC2577796

[B31] Li Z. Guo X. Huang H. Wang C. Yang F. Zhang Y. (2020). A switch in tissue stem cell identity causes neuroendocrine tumors in *Drosophila* gut. *Cell Rep.* 30 1724–1734.e4. 10.1016/j.celrep.2020.01.041 32049006

[B32] Lin M. C. Lin J. J. Hsu C. L. Juan H. F. Lou P. J. Huang M. C. (2017). GATA3 interacts with and stabilizes HIF-1α to enhance cancer cell invasiveness. *Oncogene* 36 4243–4252. 10.1038/onc.2017.8 28263977 PMC5537608

[B33] Liu L. Ding C. Fu T. Feng Z. Lee J. E. Xiao L. (2020). Histone methyltransferase MLL4 controls myofiber identity and muscle performance through MEF2 interaction. *J. Clin. Invest.* 130 4710–4725. 10.1172/JCI136155 32544095 PMC7456251

[B34] Martinez Barbera J. P. Clements M. Thomas P. Rodriguez T. Meloy D. Kioussis D. (2000). The homeobox gene Hex is required in definitive endodermal tissues for normal forebrain, liver and thyroid formation. *Development* 127 2433–2445. 10804184 10.1242/dev.127.11.2433

[B35] Mauffrey P. Tchitchek N. Barroca V. Bemelmans A. Firlej V. Allory Y. (2019). Progenitors from the central nervous system drive neurogenesis in cancer. *Nature* 569 672–678. 10.1038/s41586-019-1219-y 31092925

[B36] Melvin V. S. Feng W. Hernandez-Lagunas L. Artinger K. B. Williams T. (2013). A morpholino-based screen to identify novel genes involved in craniofacial morphogenesis. *Dev. Dyn.* 242 817–831. 10.1002/dvdy.23969 23559552 PMC4027977

[B37] Monaghan A. P. Kaestner K. H. Grau E. Schütz G. (1993). Postimplantation expression patterns indicate a role for the mouse forkhead/HNF-3 alpha, beta and gamma genes in determination of the definitive endoderm, chordamesoderm and neuroectoderm. *Development* 119 567–578. 8187630 10.1242/dev.119.3.567

[B38] Motallebipour M. Ameur A. Reddy Bysani M. S. Patra K. Wallerman O. Mangion J. (2009). Differential binding and co-binding pattern of FOXA1 and FOXA3 and their relation to H3K4me3 in HepG2 cells revealed by ChIP-seq. *Genome Biol.* 10:R129. 10.1186/gb-2009-10-11-r129 19919681 PMC3091322

[B39] Pan J. Silva T. C. Gull N. Yang Q. Plummer J. T. Chen S. (2020). Lineage-specific epigenomic and genomic activation of oncogene HNF4A promotes gastrointestinal adenocarcinomas. *Cancer Res.* 80 2722–2736. 10.1158/0008-5472.CAN-20-0390 32332020

[B40] Parang B. Barrett C. W. Williams C. S. (2016). AOM/DSS model of colitis-associated cancer. *Methods Mol. Biol.* 1422 297–307. 10.1007/978-1-4939-3603-8_26 27246042 PMC5035391

[B41] Partridge E. C. Chhetri S. B. Prokop J. W. Ramaker R. C. Jansen C. S. Goh S. T. (2020). Occupancy maps of 208 chromatin-associated proteins in one human cell type. *Nature* 583 720–728. 10.1038/s41586-020-2023-4 32728244 PMC7398277

[B42] Peng K. Kou L. Yu L. Bai C. Li M. Mo P. (2019). Histone Demethylase JMJD2D interacts with β-catenin to induce transcription and activate colorectal cancer cell proliferation and tumor growth in mice. *Gastroenterology* 156 1112–1126. 10.1053/j.gastro.2018.11.036 30472235

[B43] Polyak S. Mach A. Porvasnik S. Dixon L. Conlon T. Erger K. E. (2012). Identification of adeno-associated viral vectors suitable for intestinal gene delivery and modulation of experimental colitis. *Am. J. Physiol. Gastrointest. Liver Physiol.* 302 G296–G308. 10.1152/ajpgi.00562.2010 22114116

[B44] Qu C. Zheng D. Li S. Liu Y. Lidofsky A. Holmes J. A. (2018). Tyrosine kinase SYK is a potential therapeutic target for liver fibrosis. *Hepatology* 68 1125–1139. 10.1002/hep.29881 29537660 PMC6138581

[B45] Ralston A. Cox B. J. Nishioka N. Sasaki H. Chea E. Rugg-Gunn P. (2010). Gata3 regulates trophoblast development downstream of Tead4 and in parallel to Cdx2. *Development* 137 395–403. 10.1242/dev.038828 20081188

[B46] Rubin H. (1985). Cancer as a dynamic developmental disorder. *Cancer Res.* 45 2935–2942. 3891078

[B47] Saha S. K. Parachoniak C. A. Ghanta K. S. Fitamant J. Ross K. N. Najem M. S. (2014). Mutant IDH inhibits HNF-4α to block hepatocyte differentiation and promote biliary cancer. *Nature* 513 110–114. 10.1038/nature13441 25043045 PMC4499230

[B48] Seiliez I. Thisse B. Thisse C. (2006). FoxA3 and goosecoid promote anterior neural fate through inhibition of Wnt8a activity before the onset of gastrulation. *Dev. Biol.* 290 152–163. 10.1016/j.ydbio.2005.11.021 16364286

[B49] Sipos F. Firneisz G. Mũzes G. (2016). Therapeutic aspects of c-MYC signaling in inflammatory and cancerous colonic diseases. *World J. Gastroenterol.* 22 7938–7950. 10.3748/wjg.v22.i35.7938 27672289 PMC5028808

[B50] Soufi A. Jayaraman P. S. (2008). PRH/Hex: an oligomeric transcription factor and multifunctional regulator of cell fate. *Biochem. J.* 412 399–413. 18498250 10.1042/BJ20080035 PMC2570084

[B51] Subramanian A. Tamayo P. Mootha V. K. Mukherjee S. Ebert B. L. Gillette M. A. (2005). Gene set enrichment analysis: a knowledge-based approach for interpreting genome-wide expression profiles. *Proc. Natl. Acad. Sci. U.S.A.* 102 15545–15550. 10.1073/pnas.0506580102 16199517 PMC1239896

[B52] Takaku M. Grimm S. A. Wade P. A. (2015). GATA3 in breast cancer: tumor suppressor or oncogene? *Gene Expr.* 16 163–168. 10.3727/105221615X14399878166113 26637396 PMC4758516

[B53] Takashima Y. Horisawa K. Udono M. Ohkawa Y. Suzuki A. (2018). Prolonged inhibition of hepatocellular carcinoma cell proliferation by combinatorial expression of defined transcription factors. *Cancer Sci.* 109 3543–3553. 10.1111/cas.13798 30220099 PMC6215883

[B54] Tanaka M. Lyons G. E. Izumo S. (1999). Expression of the Nkx3.1 homobox gene during pre and postnatal development. *Mech. Dev.* 85 179–182. 10.1016/s0925-4773(99)00084-2 10415359

[B55] Thisse B. Pflumio S. Fürthauer M. Loppin B. Heyer V. Degrave A. (2001). *Expression of the Zebrafish Genome During Embryogenesis (NIH R01 RR15402). ZFIN Direct Data Submission.* Available online at: http://zfin.org (accessed August 20, 2020).

[B56] Walesky C. Apte U. (2015). Role of hepatocyte nuclear factor 4α (HNF4α) in cell proliferation and cancer. *Gene Expr.* 16 101–108. 10.3727/105221615X14181438356292 25700366 PMC5841246

[B57] Walmsley M. Ciau-Uitz A. Patient R. (2002). Adult and embryonic blood and endothelium derive from distinct precursor populations which are differentially programmed by BMP in *Xenopus*. *Development* 129 5683–5595. 10.1242/dev.00169 12421708

[B58] Wang Y. Zhang J. Wang Y. Wang S. Zhang Y. Miao Q. (2019). Expression status of GATA3 and mismatch repair proteins in upper tract urothelial carcinoma. *Front. Med.* 13:730–740. 10.1007/s11684-019-0687-7 31020542

[B59] Xicoy H. Wieringa B. Martens G. J. (2017). The SH-SY5Y cell line in Parkinson’s disease research: a systematic review. *Mol. Neurodegener.* 12:10. 10.1186/s13024-017-0149-0 28118852 PMC5259880

[B60] Xu L. Zhang M. Shi L. Yang X. Chen L. Cao N. (2021). Neural stemness contributes to cell tumorigenicity. *Cell Biosci.* 11:21. 10.1186/s13578-021-00531-6 33468253 PMC7814647

[B61] Yachida S. Fukushima N. Nakanishi Y. Akasu T. Kitamura H. Sakamoto M. (2003). Alpha-fetoprotein-producing carcinoma of the colon: report of a case and review of the literature. *Dis. Colon. Rectum.* 46 826–831. 10.1007/s10350-004-6663-5 12794586

[B62] Yahoo N. Pournasr B. Rostamzadeh J. Hakhamaneshi M. S. Ebadifar A. Fathi F. (2016). Enforced expression of Hnf1b/Foxa3 promotes hepatic fate of embryonic stem cells. *Biochem. Biophys. Res. Commun.* 474 199–205. 10.1016/j.bbrc.2016.04.102 27107701

[B63] Yin C. Lin Y. Zhang X. Chen Y. X. Zeng X. Yue H. Y. (2008). Differentiation therapy of hepatocellular carcinoma in mice with recombinant adenovirus carrying hepatocyte nuclear factor-4alpha gene. *Hepatology* 48 1528–1539. 10.1002/hep.22510 18925631

[B64] Zaki M. H. Vogel P. Body-Malapel M. Lamkanfi M. Kanneganti T. D. (2010). IL-18 production downstream of the Nlrp3 inflammasome confers protection against colorectal tumor formation. *J. Immunol.* 185 4912–4920. 10.4049/jimmunol.1002046 20855874 PMC3104023

[B65] Zhang Z. Lei A. Xu L. Chen L. Chen Y. Zhang X. (2017). Similarity in gene-regulatory networks suggests that cancer cells share characteristics of embryonic neural cells. *J. Biol. Chem.* 292 12842–12859. 10.1074/jbc.M117.785865 28634230 PMC5546026

