# Supplementary material for: Suppression of Cell Tumorigenicity by Non-neural Pro-differentiation Factors via Inhibition of Neural Property in Tumorigenic Cells
Source: Front Cell Dev Biol. 2021 Sep 14;9:714383. doi: 10.3389/fcell.2021.714383 (PMC8476888; doi:10.3389/fcell.2021.714383)
Supplement: Supplementary file 1 [file Presentation_1.pdf]

SUPPLEMENTARY MATERIAL

Supplementary Figures

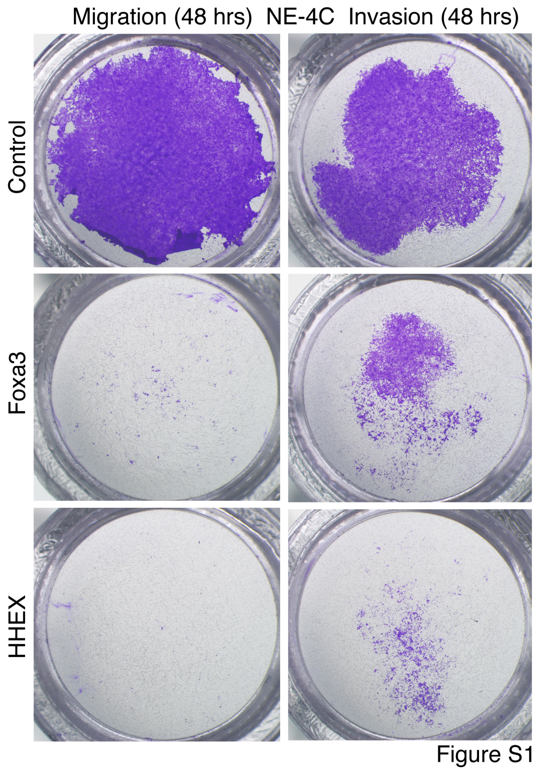

Supplementary Figure 1. Effect of Foxa3 or HHEX on the migration and invasion capability of NE-4C cells.

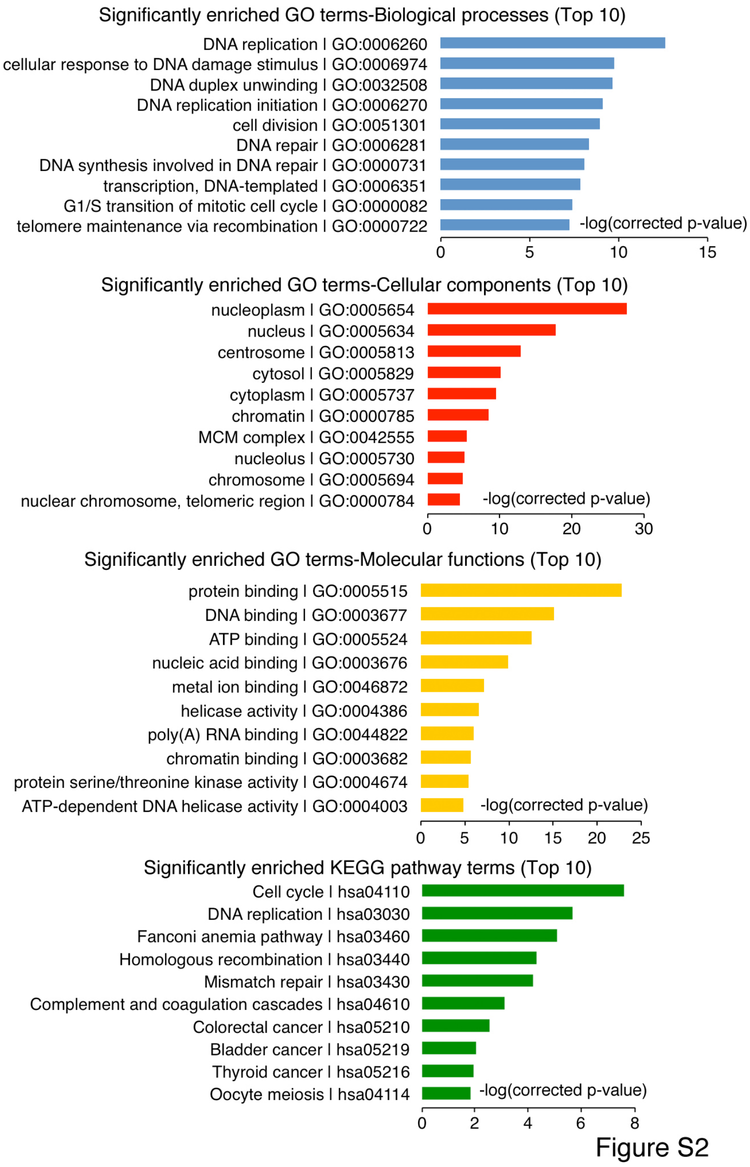

Supplementary Figure 2. Enrichment analysis on differentially expressed genes in response to FOXA3 overexpression in HepG2 cells.

A

HHEX binding sites

SOX2 promoter (-373/+43)

```
cgcgtcccatcctcatttaagtaccctgcaccaaaaagtaaatcaatattaagtttaaagaa
aaaaaaacccacgtagtcttagtgctgtttacccacttccttcgaaaaggcgtgtggtgtga
cctgttgctgcgagaggggatacaaaggtttctcagtggctggcaggctggctctgggagcc
tcctccccctcctcgcctgcccccctcctcccccggcctcccccgcgcggccggcggcgcggg
aggccccgccccctttcatgcaaaacccggcagcgaggctgggctcgagtggaggagccgcc
gcgcgctgattggtcgctagaaacccatttattccctgacagcccccgtcacatggatggtt
gtc(+1)tattaacttgttcaaaaaagtatcaggagttgtcaaggcaga
```

CDKN1A promoter (-898/+61)

```
ccatgcccggctgattttgtatttttaatggagacggggtttcaccatattggccaggctg
gtctcaaaactcctgaccctgtgatctgcccgcctcggcctcccaaagtgctgggattacag
gcgtaagccaccacgcccggccagtatatatttttaattgagaagcaaaattgtacttcaga
tttgtgatgctaggaacatgagcaaactgaaaattactaaccacttgtcagaaacaataaat
ccaactttttgtgcaaaaaaaaaaaatacaaatattagctgggcatggtggtgcatgcctgta
atcccagctactcgggaggctgaggcagaattgcttgaacctgggaggcggagactgcagtg
agctgagattgtgccactgctgactttgtctcaaaaaacaaaacaaaacaaaaaaacaaaat
gaaaacaaaaagccagggctgcctctgctcaataatgttctatctttgttccgcctcttctc
tggggtctcacttcttgggagcctgtgtgaaggtgaattcctctgaaagctgactgcccc ta
tttgggactccccagtctctttctgagaaatggtgacattgttcccagcacttcctctccct
tcctaggcagcttctgcagccaccactgagccttcctcacatcctccttcttcaggcttggg
ctttccacctttcaccattcccctaccccatgctgctccaccgcactctggggagggggctg
gactgggcactcttgtcccccaggctgagcctccctccatccctatgctgcctgcttcccag
gaacatgcttgggcagcaggctgtggctctgattggctttctggccgtcaggaacatgtccc
aacatgttgagctctggcatagaagaggctg(+1)gtggctatttgtccttgggctgcctg
ttttcaggtgaggaaggggatggtaggagacag
```

B

FOXA consensus binding sites

SOX2 promoter (-373/+43)

```
cgcgtcccatcctcatttaagtaccctgcaccaaaaagtaaatcaatattaagtttaaagaa
aaaaaaacccacgtagtcttagtgctgtttacccacttccttcgaaaaggcgtgtggtgtga
cctgttgctgcgagaggggatacaaaggtttctcagtggctggcaggctggctctgggagcc
tcctccccctcctcgcctgcccccctcctcccccggcctcccccgcgcggccggcggcgcggg
aggccccgccccctttcatgcaaaacccggcagcgaggctgggctcgagtggaggagccgcc
gcgcgctgattggtcgctagaaacccatttattccctgacagcccccgtcacatggatggtt
gtc(+1)tattaacttgttcaaaaaagtatcaggagttgtcaaggcaga
```

SBDS promoter (-470/51)

```
tgacagagtgagactgacttaaaaaaaaaactgtaaataaaaaagcaacaaaaaaatgaaaa
aagaataaaacaaggatggcaaaatgttgatagcggggtgatgggtacatagaaggttcatt
acactttttttgtctacattttatgttaaaatatttctattataaaagggtgagtaaataaaa
atatttattataaaataattctattataaaaaatatttatattataaagtgggtctggccct
tgaattccgcggaacgaggtggtgccaacgctgtgttttaacccggtcactaaacatccgcg
agcatcctgtcagagctctcagctcattggcgaaagtaaatacgccaaggaaaagcacctcc
ctttttgggcgtggaaagatggcgtaaaaagccacaatacgcaggcgtcatcgctcactttt
cccctcccggcttctgctccacctgacgcctgcgcag(+1)taagtaagcctgccagacaca
ctgtgacggctgcctgaagctagtgagtc
```

Figure S3

Supplementary Figure 3. HHEX binding sites (boxed sequences) in *SOX2* and *CDKN1A* promoters (A) and FOXA consensus binding sites (boxed sequences) in *SOX2* and *SBDS* promoters (B). Regions of promoters are indicated with numbers. +1 designates the transcription start site.

Supplementary Tables

Supplementary Table 1. Primers for RT-qPCR and ChIP-qPCR

Primers for RT-qPCR	
Mouse genes	Primers (5'>3')
β-Act	Forward: ccctgaagtaccccattgaa<br>Reverse: cttttcacggttggccttag
Ascl1	Forward: gccaacaagaagatgagcaag<br>Reverse: gaacccgccatagagttcaa
Bdnf	Forward: ggtatccaaaggccaactga<br>Reverse: cttatgaatcgccagccaat
Cdh2	Forward: cggtttcacttgagagcaca<br>Reverse: catacgtcccaggctttgat
Cdk1	Forward: ctccactccggttgacatct<br>Reverse: actcgacttctggccacact
Cdkn1a	Forward: gtccaatcctggtgatgtcc<br>Reverse: tccgtgacgaagtcaaagttc
Cenpu	Forward: aaggaccaaggtcacagcaa<br>Reverse: gccgcactttcttctgagtt
Eif1b	Forward: aggaagacgctgaccactgt<br>Reverse: gatgccaacctccagaagaa
Ezh2	Forward: gtgctgaagcctccatgttt<br>Reverse: acatcctcagtgggaacagg
Foxa3	Forward: ctcctacatgaccttgaacc<br>Reverse: aggaatatggtggtttggcg
Hes1	Forward: gaaagatagctcccggcatt<br>Reverse: gtcacctcgttcatgcactc
Lsd1	Forward: cgaatgacctctcaggaagc<br>Reverse: gctggagagtggcttcaaac
Mcm4	Forward: gggtaaattccgtgctgaga<br>Reverse: cctggtctcagggtctttca
Mcm7	Forward: taccagccaatccagtctcc<br>Reverse: cccacaggaacttggtcact
Msi1	Forward: acgtttgagagcgaggacat<br>Reverse: atacccagcatgaaggcatc
Myc	Forward: acgactccgtacagccctatt<br>Reverse: acgtagcgaccgcaacata
Nefl	Forward: ccgaagagtggttcaagagc<br>Reverse: cagctgcttctccagagctt
Pax6	Forward: cacatcaggttccatgttgg<br>Reverse: cataactccgcccattcact
Rps7	Forward: cgtggtcttcattgctcaga<br>Reverse: tcacacggatcctcttaccc
Sbds	Forward: actccgtgaaacccaacaag<br>Reverse: caccttcatcagtggcttca
Sox1	Forward: cacaactcggagatcagcaa<br>Reverse: tccttcttgagcagcgtctt
Sox2	Forward: gcggagtggaaacttttgtc<br>Reverse: tccgggaagcgtgtacttat

Srsf2	Forward: gctccagatcaacctccaag<br>Reverse: ctgctccctcttcttctgga
Srsf10	Forward: cgaagccggagttatgaaag<br>Reverse: attccagctgcagtttggtc
Tubb3	Forward: ttctggtggacttggaacct<br>Reverse: actctttccgcacgacatct
Vim	Forward: gaccttgaacggaaagtgga<br>Reverse: agccacgctttcatactgct
Zic1	Forward: tggagccttcttccgctat<br>Reverse: actcctcccagaagcagatgt
Human genes	Primers (5’>3’)
β-ACT	Forward: agaaaatctggcaccacacc<br>Reverse: tagcacagcctggatagcaa
AFP	Forward: agcttggtggtggatgaaac<br>Reverse: tctgcaatgacagcctcaag
ASCL1	Forward: gtgcgaatggactttggaag<br>Reverse: gggttggttgactgttttcg
CDH1	Forward: tggacagggaggattttgag<br>Reverse: acctgaggctttggattcct
CDH2	Forward: ccatcactcggcttaatggt<br>Reverse: acccacaatcctgtccacat
CDK1	Forward: ttttcagagctttgggcact<br>Reverse: aggcttcctggtttccattt
CDKN1A	Forward: cactcgtcaaatcctcccctt<br>Reverse: tccagtggtgtctcggtga
CENPF	Forward: cacaagccaccatgaatcac<br>Reverse: gtcacctcttgttccccaga
CENPU	Forward: agtttgtaaggcagccatcg<br>Reverse: tggctctaaccgaagcagtt
DNMT1	Forward: gagccacagatgctgacaaa<br>Reverse: tgccattaacaccaccttca
EZH2	Forward: agggcacagcagaagaactaa<br>Reverse: cgcctacagaaaagcgtatga
FOXM1	Forward: tgctagctgaggaggggata<br>Reverse: atgggtctcgctaagtgtgg
GAPDH	Forward: tcaagaaggtggtgaagcag<br>Reverse: tgacaaagtggtcgttgagg
HHEX	Forward: acccgacgcccttttacat<br>Reverse: aagaaggggctccagagtaga
MCM4	Forward: atggcggtgctaaaggacta<br>Reverse: cgagggtatgcagaaaccat
MCM7	Forward: tgaactcgggaagaagcagt<br>Reverse: tgtacggcatcagcaaagag
MSI1	Forward: accaagagatccaggggttt<br>Reverse: tcgttcgagtcaccatcttg
SBDS	Forward: ttcggttcatccttccagtc<br>Reverse: cccgggtcaatcagacatac
MYC	Forward: tcaagaggcgaacacacaac

	Reverse: atgagcttttgctcctctgc
SOX2	Forward: catcacccacagcaaatgac<br>Reverse: cctccccaggttttctctgta
SRSF10	Forward: cacgtctctgttcgtcagga<br>Reverse: ggacggcgagtgtagaaatc
Primers for ChIP-qPCR	
Gene	Primers (5’>3’. Predicted amplified regions are indicated by numbers at the end of each reverse primer. The first base of the transcriptional start site is designated as +1.)
AFP	Primer pair 1<br>Forward: cgcatagcagaaaatggaca (-1547)<br>Reverse: cgtcagtagcacttccagca (-1431)<br>primer pair 2<br>forward: aagtccccttgaggaagag (-1705)<br>Reverse: acaggccttgctcacgtact (-1589)<br>Primer pair 3<br>Forward: ttcaaaaagttcccccagtg (-868)<br>Reverse: aagtgggtcaggtgcatcat (-715)<br>Primer pair 4<br>Forward: ttgcataccaaatctgctgtct (-350)<br>Reverse: agctctttggggcagaaaa (-169)
CDKN1A	Primer pair 1<br>Forward: ggctggtctcaaaactcctg (-841)<br>Reverse: gctcatgttcctagcatcac (-689)<br>Primer pair 2<br>Forward: tctcagctcactgcaacctc (-958)<br>Reverse: tggtggcttacgcctgtaat (-761)
EZH2	Primer pair 1<br>Forward: gcttgcagtgagctgagatg (-1811)<br>Reverse: tcacctatgaactgttggggta (-1656)
FOXM1	Primer pair 1<br>Forward: tcgtgacctcaagtgatcca (-799)<br>Reverse: tggcagacaaggttctttcc (-637)
MYC	Primer pair 1<br>Forward: ccgcctgcgatgatttatac (-504)<br>Reverse: cgcctaccattttcttttgc (-387)<br>primer pair 2<br>Forward: tattcataacgcgctctcca (-790)<br>Reverse: cagggagagtggaggaaaga (-622)<br>Primer pair 3<br>Forward: gggaaagaggacctggaaag (-1373)<br>Reverse: gggaccggacttcctaaaag (-1231)
SBDS	Primer pair 1<br>Forward: tcagctcattggcgaaagta(-142)<br>Reverse: cagtgtgtctggcaggctta (+25)<br>Primer pair 2<br>Forward: aaatgttgatagcggggtga(-387)<br>Reverse: aagggccagacccactttat(-221)

	Primer pair 3<br>Forward: aagctaacgctggaagatcg(-553)<br>Reverse: catcaccccgctatcaacat(-365)
SOX2	Primer pair 1<br>Forward: gcgtcccatcctcatttaag (-372)<br>Reverse: agcaacaggtcacaccacac (-241)

Supplementary Table 6. Xenograft analysis on different types of cells

Cell type	Treatment	Cells injected per mouse	Days after injection	Tumors/ Injection
NE-4C	Vector (puro)	1×10^6	31	3/3
	HHEX (puro)			0/4
NE-4C	Vector (puro)	2×10^6	31	4/4
	Foxa3 (puro)			3/3
HCT116	Vector (puro)	5×10^6	29	8/8
	GATA3 (puro)			5/8
HCT116	Vector (puro)	5×10^6	25	4/4
	Hnf4a (puro)			1/4
HCT116	Vector (puro)	5×10^6	29	5/5
	HHEX (puro)			5/5
	Foxa3 (puro)			5/5
	HHEX+Foxa3 (puro)			5/5
HCT116 (sorted)	Vector (GFP)	2.5×10^6	31	8/8
	Foxa3 (GFP)			0/8
HCT116 (Sorted and non-infected cell mix, 2:1)	Vector (GFP)/ non-infected cell mix	2.5×10^6	31	7/7
	Foxa3 (GFP)/ non-infected cell mix			5/7
HCT116	Vector (GFP)	2.5×10^6	26	5/5
	HHEX (GFP)			3/5

Supplementary Table 2. Gene associated peaks representing FOXA3 binding sites derived from ChIP-seq data from HepG2 cells with FOXA3 overexpression.

Supplementary Table 3. Differentially expressed genes (DEGs) between HepG2 with FOXA3 overexpression and control cells with expression of EGFP.

Supplementary Table 4. Putative target genes of FOXA3.

Supplementary Table 5. HHEX target genes. The dataset was downloaded from the Molecular Signatures Database (MSigDB) (www.gsea-msigdb.org/gsea/msigdb/cards/HHEX_TARGET_GENES.html)
